# Mechanistic Studies of Iron-PyBOX-Catalyzed Olefin Amino-Oxygenation with Functionalized Hydroxylamines

**DOI:** 10.1021/acs.organomet.3c00067

**Published:** 2023-04-28

**Authors:** Aleksa Radović, Nikki J. Wolford, Hongze Li, William W. Brennessel, Hao Xu, Michael L. Neidig

**Affiliations:** † Department of Chemistry, University of Rochester, Rochester, New York 14627, USA; ‡ Department of Chemistry, Brandeis University, Waltham, Massachusetts 02453, USA

## Abstract

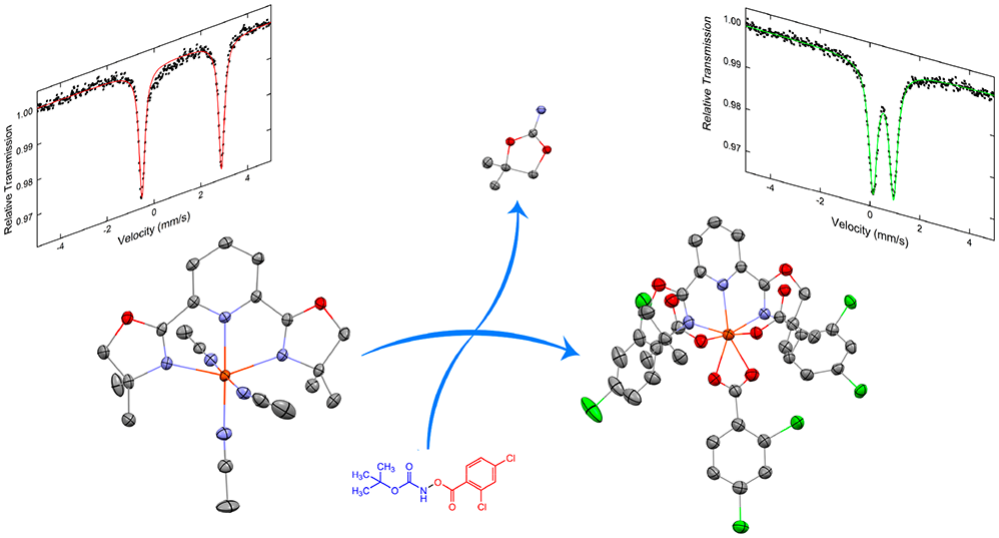

Iron-catalyzed amino-oxygenation of olefins often uses discrete ligands to increase reactivity and broaden substrate scope. This work is focused on examining ligand effects on reactivity and in situ iron speciation in a system which utilizes a bisoxazoline ligand. Freeze-trapped ^57^Fe Mössbauer and EPR spectroscopies as well as SC-XRD experiments were utilized to isolate and identify the species formed during the catalytic reaction of amino-oxygenation of olefins with functionalized hydroxylamines, as well as in the precatalytic mixture of iron salt and ligand. Experiments revealed significant influence of ligand and solvent on the speciation in the precatalytic mixture which led to the formation of different species which had significant influence on the reactivity. In situ experiments showed no evidence for the formation of an Fe(IV)-nitrene intermediate, and the isolation of a reactive intermediate was unsuccessful, suggesting that the use of the PyBOX ligand led to the formation of more reactive intermediates than observed in the previously studied system, preventing direct detection of intermediate species. However, isolation of the seven coordinate Fe(III) species with three carboxylate units of the hydroxylamine and spin-trap EPR experiments suggest formation of a species with unpaired electron density on the hydroxylamine nitrogen, which is in accordance with formation of a potential iron iminyl radical species, as recently proposed in literature. An observed increase in yield when substrates devoid of C–H bonds as well as isolation of a ring-closed dead-end species with substrates containing these bonds suggests the identity of the functionalized hydroxylamine can dictate the reactivity observed in these reactions.

## 1 Introduction

Catalytic alkene difunctionalization is a powerful strategy for the rapid assembly of complex organic molecules with a wide range of applications including the pharmaceutical and agricultural industries.^1^ In particular, 1,2-amino-oxygenation reactions are of interest as a simple method to introduce both nitrogen and oxygen into a molecule with a single step. The first example of these 1,2-amino-oxygenation reactions was reported by Sharpless and involves the use of an osmium catalyst and chloramine-T as the nitrogen source.^2,3^ These initial efforts toward 1,2-amino-oxygenation reactions have inspired the development of ulterior approaches which allow for broader substrate scopes and better regioselectivity^4−15^ and employ nonprecious metal catalysts in the form of iron or copper.^16−23^

**Scheme 1:**
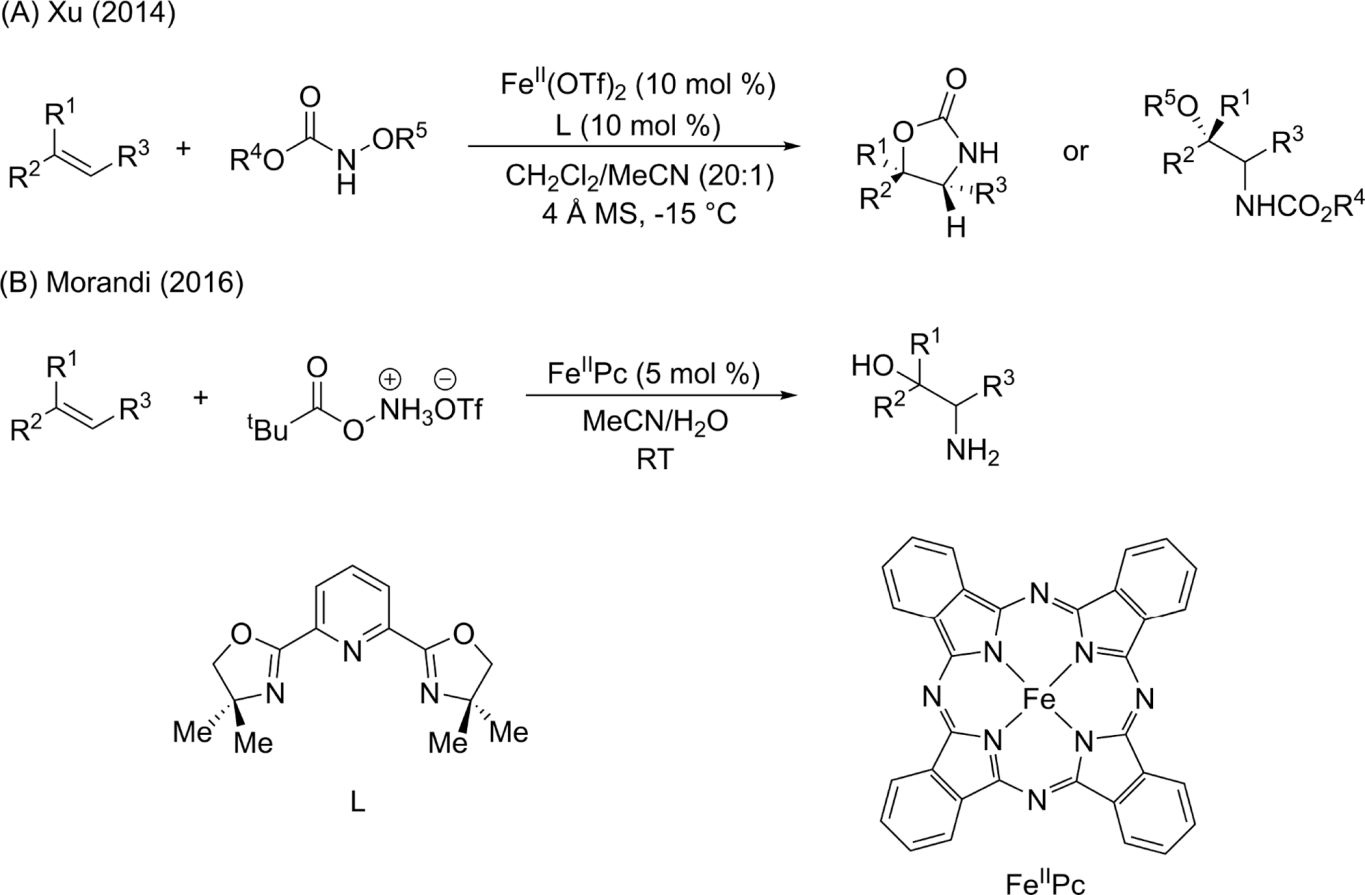
Iron-Catalyzed Intermolecular Amino Oxygenation of Olefins (A) With functional hydroxylamines^27^ and (B) with acyloxy aminium triflate.^28^

In addition to diversifying the catalyst employed in these methods, the use of different nitrogen sources, such as hydroxylamine derivatives, have been investigated as well.^24^ The first example of a base-metal-catalyzed olefin amino-oxygenation which utilized hydroxylamines as the nitrogen source was reported by Xu and co-workers in 2013.^25,26^ This iron-catalyzed method for intramolecular olefin aminohydroxylation utilized bidentate nitrogen-based ligands and simple iron salts to form amino alcohols with high selectivity. Following this discovery, Xu and co-workers expanded this method to include iron-catalyzed intermolecular olefin amino-oxygenation method which utilized simple iron salts and nitrogen-based tridentate ligands (Scheme 1A).^27^ In 2016, Morandi and Legnani reported a method for aminohydroxylation of olefins to directly afford unprotected 1,2-amino alcohols using Fe^II^ phthalocyanine complex as catalyst (Scheme 1B).^28^

Despite the significant advances in the development of effective synthetic methods for iron-catalyzed alkene difunctionalizations including alkene amino-oxygenation, the nature of the key reactive iron species and the underlying mechanism of catalysis remains largely unknown. In particular, while the formation of a reactive iron nitrene or iron iminyl radical intermediate has been proposed across these systems, experimental studies to evaluate the formation and nature of such potential intermediates are limited. A recent advancement in this area was reported by DeBeer, Neese, Morandi, and co-workers. This study utilized a multispectroscopic approach to identify the intermediates formed in the Fe(acac)_2_(H_2_O)_2_-catalyzed aminofunctionalization of styrene using an acyloxy aminium triflate under “ligand-free” conditions.^29^ Based on these spectroscopic studies, the authors proposed that the reaction proceeds through formation of an iron-bound acyloxy aminium triflate species which undergoes homolytic N–O bond cleavage to form an iron iminyl radical intermediate.

Mechanistic insight into iron-catalyzed organic transformations has shown that the introduction of a ligand can greatly affect the iron speciation present under catalytically relevant conditions. With this in mind, this study focuses on the aforementioned intermolecular amino-oxygenation method reported by Xu and co-workers to evaluate the iron species present during catalysis in the presence of a tridentate nitrogen ligand. It has been previously proposed for this reaction system that reaction of the iron–ligand complex with functionalized hydroxylamines results in formation of an iron nitrene intermediate which undergoes radical addition with olefins followed by intramolecular carboxylate–ligand transfer to afford the amino-oxygenation product;^27^ however, the exact nature of the in situ formed iron species remained unknown. While a variety of iron salts, ligands, and hydroxylamines were examined in the initial method development, this work focuses on the use of Fe(OTf)_2_ with the tridentate bisoxazoline PyBOX ligand and *tert*-butyl(2,4-dichlorobenzoyl)oxycarbamate and 2,2,2-trifluoroethyl (2,4-dichlorobenzoyl)oxycarbamate as the functionalized hydroxylamines for the amino-oxygenation of styrene. The application of an array of techniques, including freeze-trapped ^57^Fe Mössbauer and electron paramagnetic resonance (EPR) spectroscopies as well as single crystal X-ray diffraction (SC-XRD) has provided direct insight into in situ iron speciation and reactivity in olefin amino-oxygenation and expands insight into novel reactivity of the iron(II)-PyBOX complexes.

## 2 Results and Discussion

### 2.1 Iron Speciation in the Precatalytic Mixture

**Figure 1:**
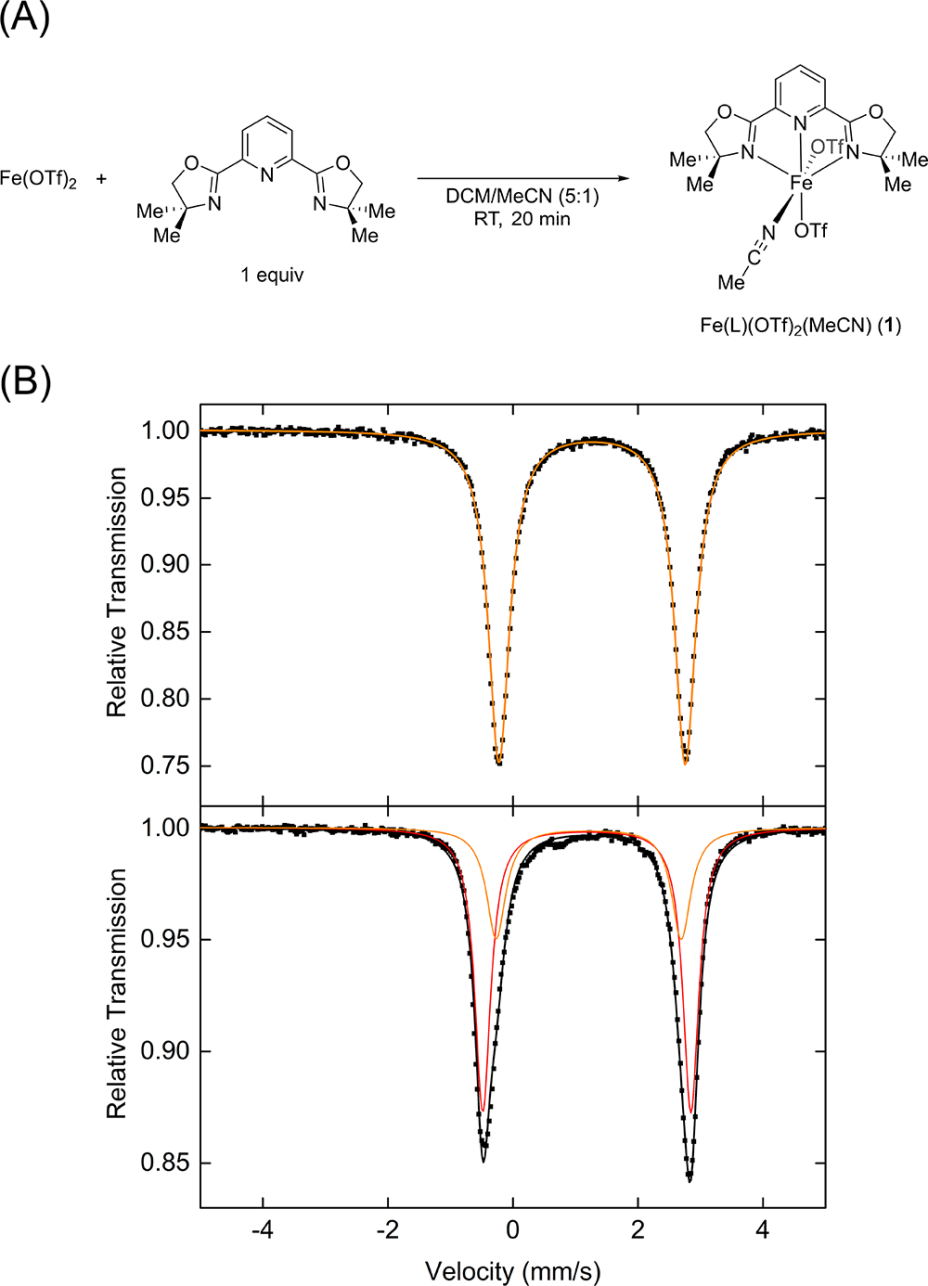
(A) Synthesis of [Fe(L)(OTf)_2_(MeCN)] (**1**). (B) (top) 80 K ^57^Fe Mössbauer spectrum of crystalline complex **1**, (bottom) 80 K ^57^Fe Mössbauer spectrum of frozen solution of complex **1** in MeCN (black line, total fit; red and orange lines, individual components).

In order to identify the iron species observed in situ under catalytically relevant conditions, initial studies were focused on the precatalytic mixture of Fe(OTf)_2_ and the PyBOX ligand. Reaction of Fe(OTf)_2_ and 1 equiv of the PyBOX ligand in a mixture of dichloromethane (DCM) and acetonitrile (MeCN) (5:1) resulted in the formation of a light red colored solution (Figure 1A). Slow evaporation of diethyl ether at −30 °C yielded red crystals. Characterization by SC-XRD revealed the formation of an Fe(II) complex bearing one PyBOX ligand, two triflates and one MeCN (complex **1**, [Fe(L)(OTf)_2_(MeCN)]), which was previously reported in the literature.^30^ The ^57^Fe Mössbauer spectrum of the crystalline material showed that complex **1** is characterized by the Mössbauer parameters δ = 1.24 mm/s and |Δ*E*_Q_| = 2.93 mm/s (Figure 1B, top), consistent with a high-spin Fe(II) complex. The frozen solution Mössbauer spectrum of crystalline **1** redissolved in MeCN was collected to determine potential solvent ligand exchange effects. This spectrum showed a slightly broadened and asymmetric signal (compared to the solid state) that can be fit to two species. One of the species corresponds to complex **1** (32% of total iron), while the other species represents an additional high-spin iron(II) species (68% of total iron), with similar isomer shift δ = 1.18 mm/s, and slightly higher quadrupole splitting than complex **1**, |Δ*E*_Q_| = 3.33 mm/s (Figure 1, bottom). This result demonstrates the effects of solvent which can lead to the formation of multiple high-spin Fe(II)-PyBOX species in the precatalytic mixture.

**Figure 2:**
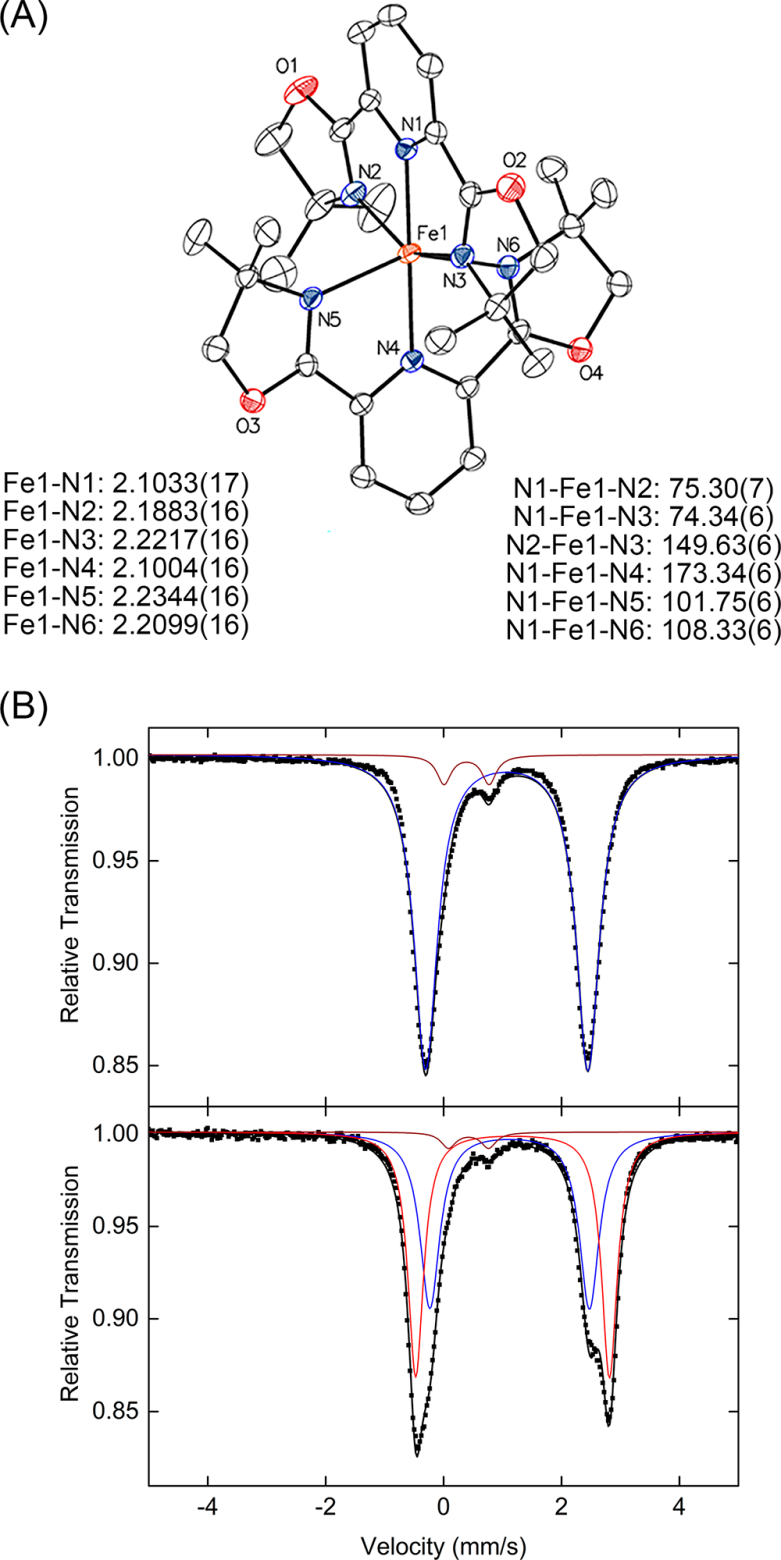
(A) Crystal structure of complex **2**. Hydrogen atoms and counterions are omitted for clarity and the thermal ellipsoids are shown at 50% probability. (B) (top) 80 K ^57^Fe Mössbauer spectrum of crystalline complex **2**, (bottom) 80 K ^57^Fe Mössbauer spectrum of frozen solution of complex **2** in MeCN (black line, total fit; blue, red, and dark red lines, individual components).

In addition to MeCN/OTf ligand exchange, the possibility of the formation of an iron species with two PyBOX ligands was also explored. The reaction of Fe(OTf)_2_ with 2 equiv of ligand in DCM resulted in a dark red solution. Slow evaporation of diethyl ether at −30 °C led to the isolation of dark red crystals. Characterization by SC-XRD revealed the formation of the targeted Fe(II) complex bearing two PyBOX ligands, with two triflate counterions (complex **2**, [Fe(L)_2_]^2+^, Figure 2A). The ^57^Fe Mössbauer spectrum of crystalline material yielded parameters of δ = 1.07 mm/s and |Δ*E*_Q_| = 2.75 mm/s (Figure 2B, top), consistent with a high-spin Fe(II) complex.

The frozen solution spectrum of crystalline material in MeCN, however, revealed the presence of an additional high-spin Fe(II) species with Mössbauer parameters consistent with the species observed when complex **1** was dissolved in MeCN (Figure 2B, bottom). This suggests that ligand exchange occurs in solution, where one of the PyBOX ligands is exchanged with either MeCN or triflate anion ligands. Additionally, a small amount (less than 5% of total iron present) of Fe(III) impurity, based on Mössbauer parameters, can be observed in both solid and solution spectrum.

**Figure 3:**
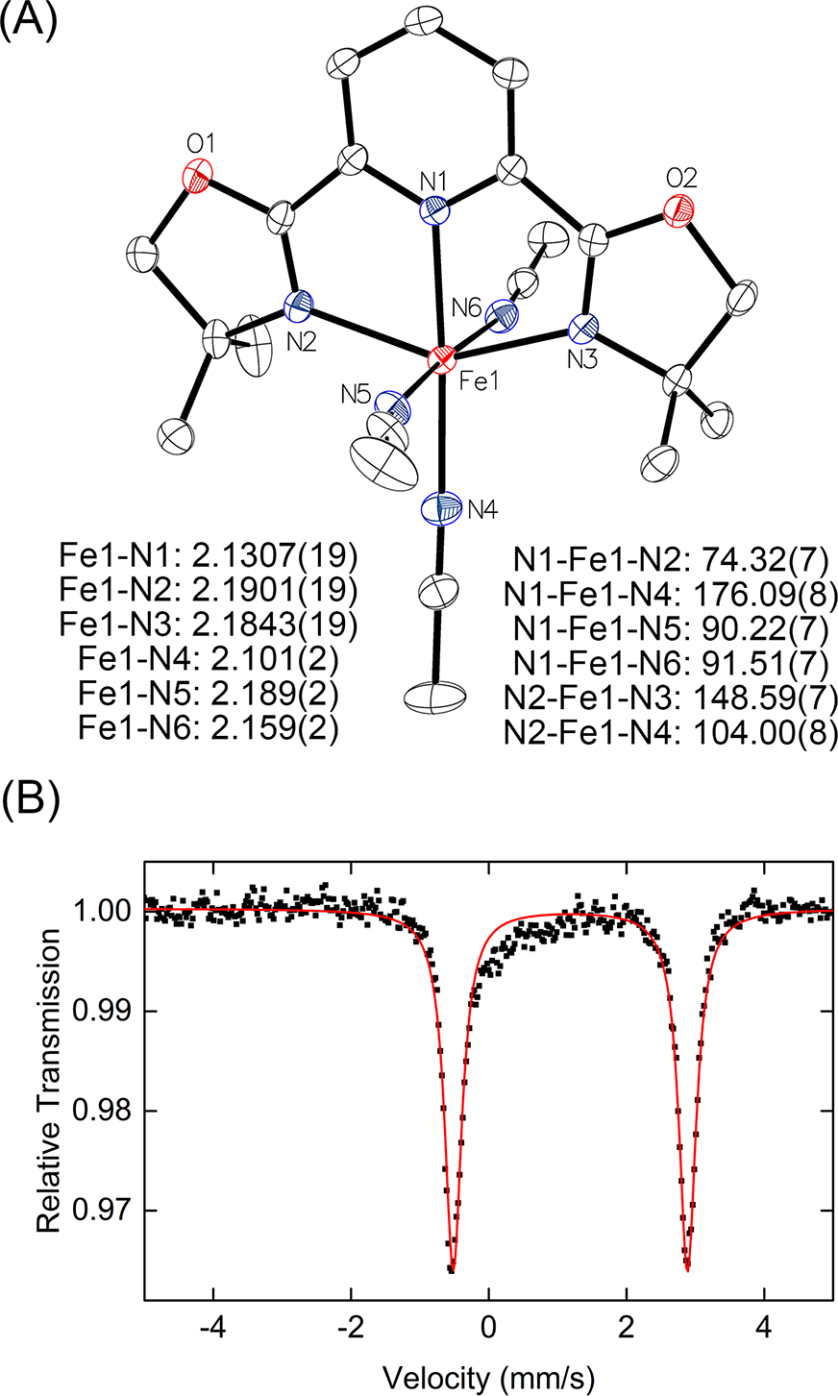
(A) Crystal structure of complex **3**. Hydrogen atoms and counterions are omitted for clarity and the thermal ellipsoids are shown at 50% probability. (B) 80 K ^57^Fe Mössbauer spectrum of crystalline complex **3**.

Based on these findings, and the large concentration of MeCN present under these conditions, it is likely that the observed iron speciation changes in solution are the result of MeCN solvent coordination. Upon reaction of Fe(NTf_2_)_2_ and the PyBOX ligand in MeCN, crystals of an iron complex bearing one PyBOX ligand and three MeCN ligands was isolated (**3**, [Fe(L)(MeCN)_3_]^2+^, Figure 3A). The solid state Mössbauer spectrum of this complex (Figure 3B) is consistent with the Mössbauer parameters of the species plotted in red which was observed in the frozen solution Mössbauer spectra of complexes **1** and **2**. This suggests that complex **3** is formed in situ upon dissolving complexes **1** or **2** in acetonitrile. However, due to the high solubility of this species and extremely low yield of crystalline material, any further characterization (frozen solution Mössbauer, UV–vis–NIR, reaction studies) could not be performed.

### 2.2 Reaction of Precatalytic Mixture with Acyloxy Carbamates

**Scheme 2:**
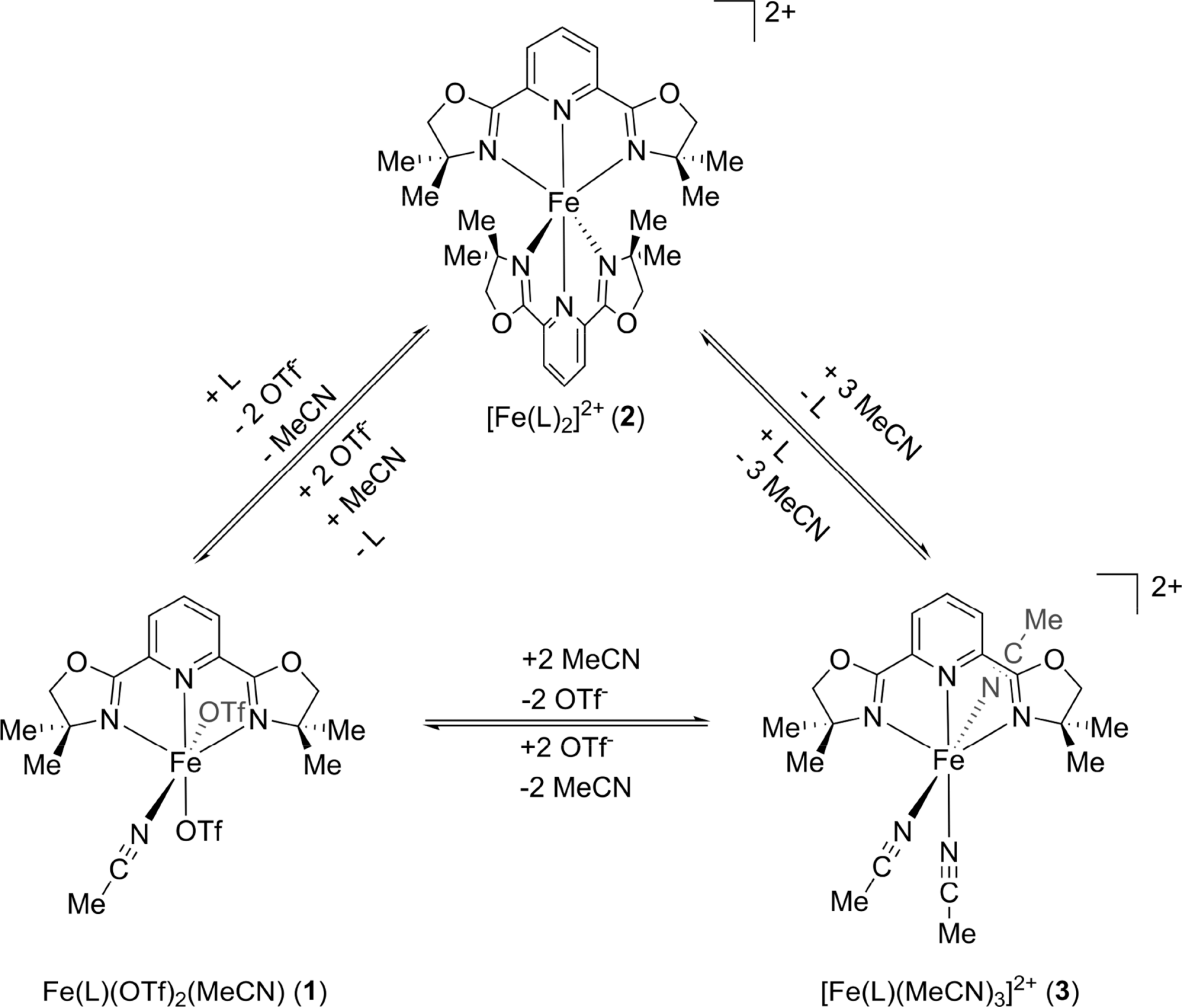
Isolated and Characterized Complexes from Precatalytic Mixture

**Figure 4:**
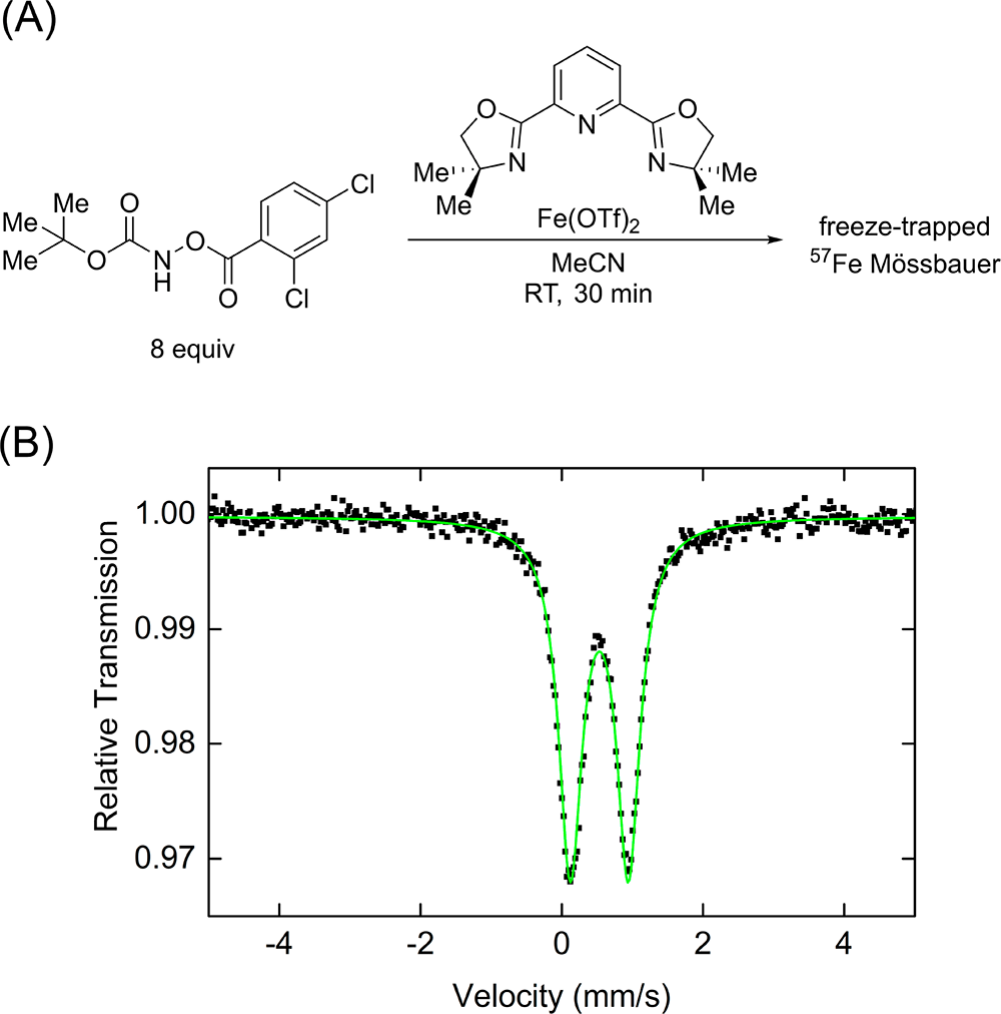
(A) Reaction between precatalytic mixture and *tert*-butyl(2,4-dichlorobenzoyl)oxycarbamate (8 equiv). (B) Freeze-trapped 80 K ^57^Fe Mössbauer spectrum of studied reaction.

Having evaluated the Fe(II) species that could be accessed upon reaction of Fe(OTf)_2_ with the PyBOX ligand (Scheme 2), the next step was to determine the reactivity of these species with acyloxy carbamates. Using ^57^Fe Mössbauer spectroscopy it was determined that reaction with excess *tert*-butyl (2,4-dichlorobenzoyl)oxycarbamate (8 equiv WRT iron) lead to the conversion of all iron species to a single species characterized with Mössbauer parameters δ = 0.53 mm/s and |Δ*E*_Q_| = 0.80 mm/s (Figure 4). The observed parameters are consistent with the Fe(III) intermediates formed in the reaction studied by DeBeer and co-workers.^10^ Additionally, the isomer shift is higher than would be expected for an Fe(IV) imido^31^ or oxo^32^ complex, further supporting formation of an Fe(III) species. To further characterize what seemed to be an Fe(III) species, the 10 K EPR spectrum was obtained of a sample prepared identically to that of the Mössbauer sample. The presence of an EPR signal from this sample further supports the assignment of an Fe(III) species over an Fe(IV) imido complex (Figure S8). Furthermore, the EPR spectrum suggested there were two unique Fe(III) species present in solution. The observation of two species in EPR spectrum and one in the Mössbauer spectrum could suggest that possibly two EPR active species have similar Mössbauer parameters that cannot be resolved in Mössbauer experiments.

To assess the reactivity of the isolated Fe(II) species, the reaction of complexes **1** and **2** with excess acyloxy carbamates, in MeCN, were performed. The ^57^Fe Mössbauer spectrum showed that the reaction of complex **1** with 8 equiv of acyloxy carbamate resulted in complete conversion of all iron species to the previously observed Fe(III) species (Figure S1). The reaction of complex **2** with 8 equiv acyloxy carbamate resulted in the formation of 46% of the Fe(III) species while 54% of the iron species remained as a high-spin Fe(II) species (Figure S2). This would suggest that there is an equilibrium between species which contain two PyBOX ligands and a species with one PyBOX ligand, which influences reactivity with the hydroxylamine. This is further supported by examining the reaction yield when starting from complexes **1** or **2** instead of the precatalytic mixture. Starting from complex **1**, a significantly higher yield is observed than when starting with complex **2** (Table S3).

**Figure 5:**
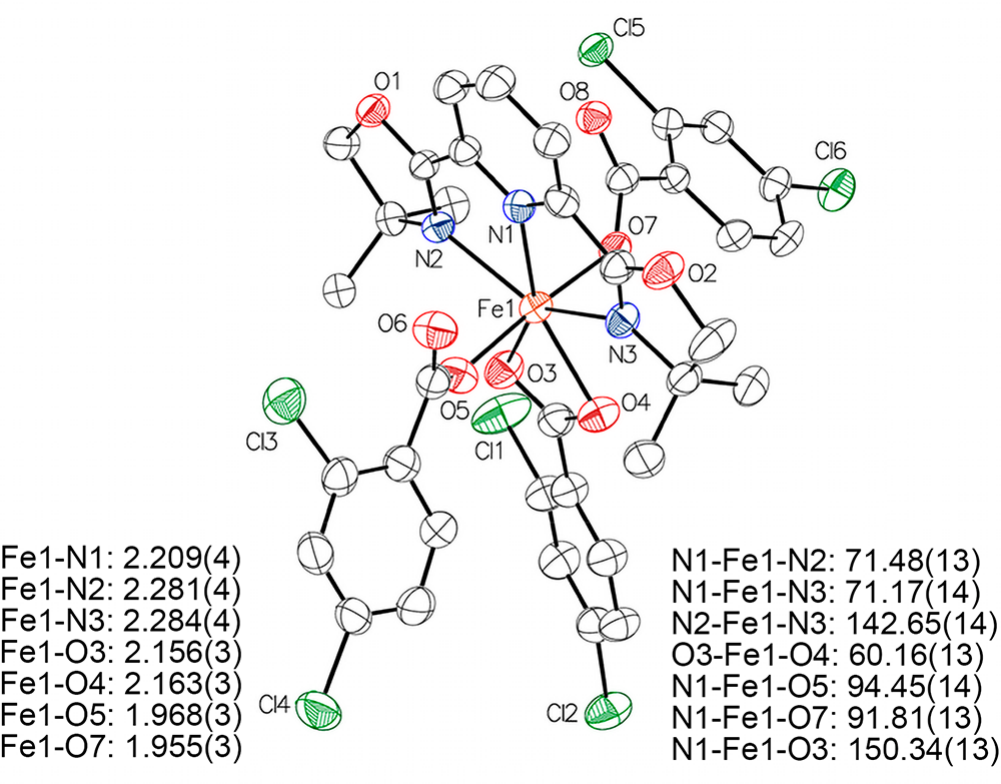
Crystal structure of complex **4**. Hydrogen atoms are omitted for clarity and the thermal ellipsoids are shown at 50% probability.

As it was shown that an Fe(III) species can be formed in situ, efforts oriented toward isolation of these species resulted in the formation of orange crystals. SC-XRD determined that crystalline material to be the seven-coordinate Fe(III) complex (**4**, [Fe(L)(DCB)_3_], DCB = 2,4-dichlorobenzoate, Figure 5). This species contains a single PyBOX ligand and three carboxylate units of the acyloxy carbamate where two carboxylates are κ^1^-coordinated and one is κ^2^ -coordinated to the iron center.

Solid state ^57^Fe Mössbauer spectroscopy showed that this species has parameters that correspond to the Mössbauer parameters of the Fe(III) species observed upon reaction of the precatalytic mixture with the acyloxy carbamate (Figure S3). Additionally, the 10 K EPR spectrum of a frozen DCM solution of this complex was consistent with a high-spin Fe(III) species as observed previously in reaction between precatalytic mixture and acyloxy carbamate (Figure S9). This is in contrast with previous work where the observed high-spin Fe(III) species was identified as an iron bound N–O species using acyloxy aminium triflate.^10^ Formation of the seven-coordinate iron complex with three carboxylates from the acyloxy carbamates suggests that this species is generated after the formation of the hypothesized iron iminyl radical species. This indicates that the proposed iron nitrogen bound intermediate is more reactive than those reported using acyloxy aminium triflate thus precluding its observation in situ. Based on these findings it can be concluded that an iron complex with a tridentate bisoxazoline PyBOX ligand leads to formation of a highly reactive iron nitrene or an iron iminyl radical species.

**Figure 6:**
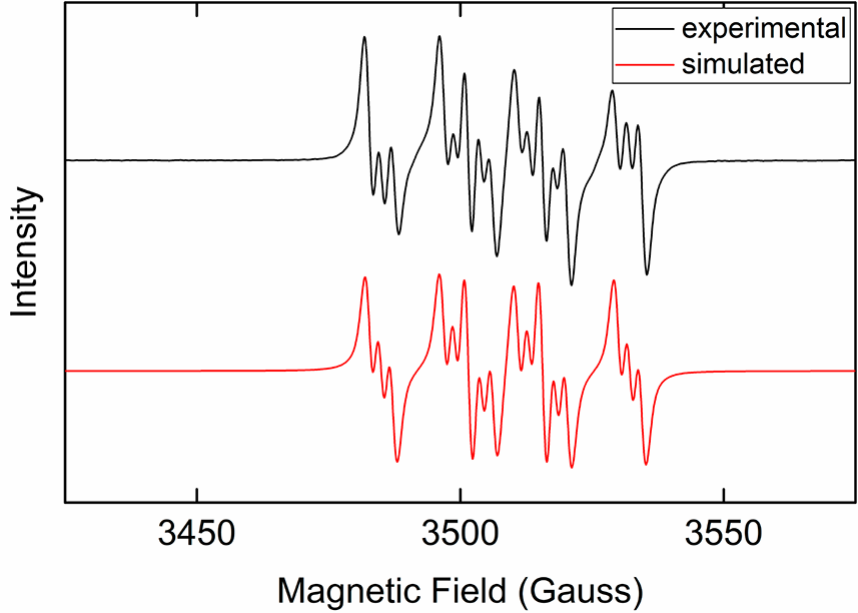
Room-temperature X-band EPR spectrum of reaction with DMPO spin-trap (black line) and simulated spectrum (red line). Simulation parameters are *g* = 2.00674, *A*_N_ = 19.0485 G, *A*_H_ = 14.0794 G, and *A*_N_ = 2.20365 G.

In the original report of this method, it was shown that reaction with a radical clock probe supported the presence of a radical species in the catalytic cycle.^27^ Initial EPR experiments did not indicate formation of a radical, which suggests a short-lived intermediate. To trap and characterize potentially formed radical species, a DMPO spin-trap was used. The control experiment of the reaction between the precatalytic mixture and DMPO spin-trap (50 equiv) showed no formation of EPR active species (Figure S11). To test the possibility of the formation of a radical species in this system, a reaction between precatalytic mixture and acyloxy carbamate (8 equiv) in the presence of DMPO (50 equiv) was studied. The room-temperature X-band EPR spectrum showed signal at *g* = 2.007 with pronounced hyperfine structure, consistent with the unpaired electron coupling to the nitrogen and hydrogen on DMPO as well as additional coupling to nitrogen, likely from the nitrogen of acyloxy carbamate (Figure 6). This suggests that the carbamate of the hydroxylamine forms a radical adduct with DMPO. From this it could be proposed that this reaction proceeds through the formation of a nitrogen centered radical (imido radical) bound to the iron center, similar to previous reports, even though it is too short-lived to be observed by freeze-trapped Mössbauer spectroscopy.

In addition to the orange crystals identified as complex **4**, additional colorless crystals were observed from the same reaction mixture. These crystals were identified by SC-XRD as the 4,4-dimethyl-1,3-dioxolan-2-iminium (**5**, see the SI). This suggests that the highly reactive radical species undergoes H-atom abstraction and subsequent C–H amination in the absence of a suitable substrate. This finding suggests that this process could be in competition with the reaction of the radical species with styrene during catalysis, which should have an impact on the reaction yield. However, this side reaction should be suppressed by exchanging the *tert*-butyl group in the hydroxylamine with trifluoroethyl or trichloroethyl groups which cannot undergo H-atom abstraction. Indeed, in the original report of this method, exchanging the *tert*-butyl group with trifluoroethyl and trichloroethyl groups resulted in higher yields for the catalytic reaction with styrene.^27^

Due to these findings, reaction of the precatalytic mixture with excess (12 equiv with regard to iron) 2,2,2-trifluoroethyl (2,4-dichlorobenzoyl)oxycarbamate was studied by ^57^Fe Mössbauer spectroscopy. The Mössbauer spectrum (Figure S5) shows formation of an Fe(III) species with parameters similar to those of complex **4**, δ = 0.52 mm/s and |Δ*E*_Q_| = 0.56 mm/s. However, in contrast to reaction with *tert*-butyl (2,4-dichlorobenzoyl)oxycarbamate, here just 32% of initial the iron species are converted to the Fe(III) species, while the rest remains as previously observed Fe(II) species. This would suggest that initial reaction of the Fe(II) species from the precatalytic mixture with 2,2,2-trifluoroethyl (2,4-dichlorobenzoyl)oxycarbamate is slower.

To confirm that the reaction with different hydroxylamine proceeds through formation of similar radical species, an analogous EPR experiment with the DMPO spin-trap was performed. Similar to the reaction with *tert*-butyl(2,4-dichlorobenzoyl)oxycarbamate, the EPR spectrum (Figure S10) shows a signal at *g* = 2.007, consistent with the unpaired electron coupling to the nitrogen and hydrogen on DMPO as well as additional coupling to nitrogen, likely from the nitrogen of acyloxy carbamate.

These findings suggest that exchanging the *tert*-butyl group with a trifluoroethyl group should prevent the formation of species which undergo competing reactions, like species **5**, while it should not influence the reaction with styrene, as both reactions proceed through formation of similar intermediates.

### 2.3 Iron Speciation in the Catalytic Reaction

Having evaluated the iron species present in situ in the precatalytic mixture, as well as in the reaction between the precatalytic mixture and acyloxy carbamate, subsequent studies focused on the elucidation of the iron species present during catalysis. Toward this aim, the catalytic reaction with Fe(OTf)_2_, PyBOX ligand, *tert*-butyl(2,4-dichlorobenzoyl)oxycarbamate, and styrene was employed as a representative example. This reaction was originally reported in a DCM/MeCN mixture (20:1) with slow addition of the precatalytic mixture to the solution of acyloxy carbamate and styrene over the course of 15 min at −15 °C. The reaction was then left to stir at this reduced temperature for an additional 45 min, giving a 48% yield of product. As chlorinated solvents are not compatible with frozen solution Mössbauer spectroscopy, the same procedure was conducted in pure MeCN to determine if the reaction would still proceed. Utilizing only MeCN as the solvent resulted in 29% yield of product. While a decrease in yield was observed, this result provided confidence for focusing our studies on the pure MeCN solvent system and allowed for the use of ^57^Fe Mössbauer spectroscopy for these studies.

**Figure 7:**
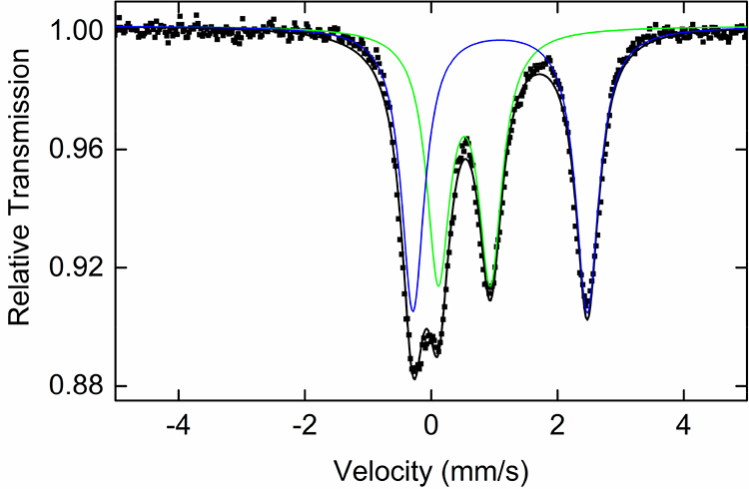
Freeze-trapped 80 K ^57^Fe Mössbauer spectrum of the catalytic reaction 15 min from starting reaction (black line, total fit; blue and green lines, individual components).

Freeze-trapped 80 K ^57^Fe Mössbauer spectroscopy of the catalytic reaction 15 min after oxidant/styrene addition revealed the presence of two iron species (Figure 7). The species plotted in green, with parameters δ = 0.53 mm/s and |Δ*E*_Q_| = 0.80 mm/s, is consistent with the formation of high-spin Fe(III) complex **4**.

**Figure 8:**
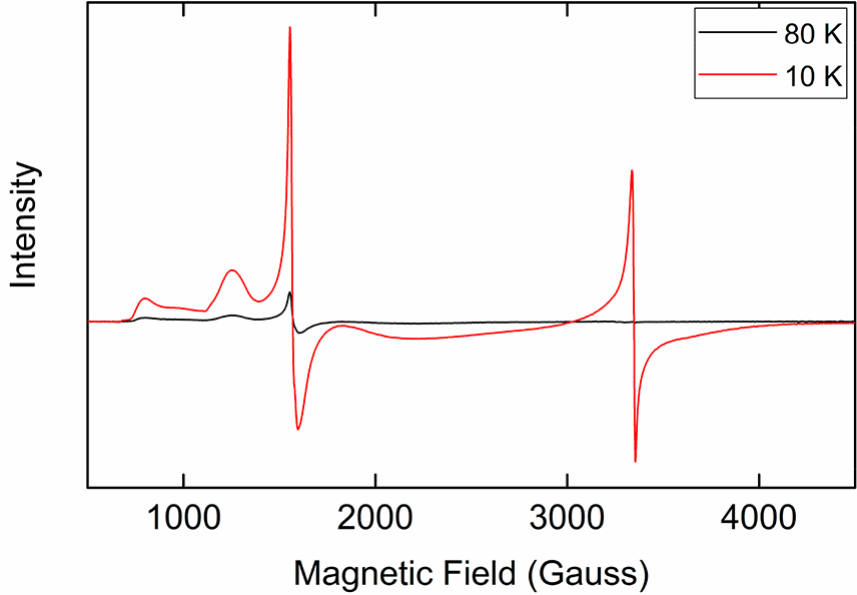
Freeze-trapped 80 K (black line) and 10 K (red line) X-band EPR spectra of catalytic reaction 15 min from starting reaction.

Formation of complex **4** in situ is further supported by 10 K X-band EPR measurements, which showed the presence of a signal with components at *g* = 4.25, 5.48, and 8.04, consistent with the EPR spectrum of isolated complex **4** (Figure 8). The second species observed by Mössbauer (blue, Figure 7) has parameters that correspond to a high-spin Fe(II) species and are in good agreement with parameters of complex **2**. However, assignment of this species as complex **2** would indicate that the high-spin Fe(III) does not have ligand bound, which is inconsistent with species that has been isolated and characterized as complex **4**. Based on this, the high-spin Fe(II) species most likely has just one PyBOX ligand. Importantly, no iron species consistent with a potential Fe(IV) nitrene species could be observed by Mössbauer spectroscopy. Lastly, monitoring the reaction at different time points showed only different ratios of the same iron components, with 62% of high-spin Fe(II) and 38% of **4** formed after 5 min compared to 42% of high-spin Fe(II) and 58% of **4** at the end of reaction (Figure S4 and Table S1).

In addition to the signals which correspond to complex **4**, the 10 K EPR spectrum of the catalytic reaction depicts the presence of an additional signal at *g* = 2.01, which corresponds to a *S* = 1/2 species. By comparing signal intensities at different temperatures, it was observed that the signal at *g* = 2.01, which corresponds to the *S* = 1/2 species, is not present at temperatures higher than 20 K, while the signal at *g* = 4.25 is present even at 100 K (Figures S12–14). This unusual behavior of the *S* = 1/2 signal suggests the presence of a magnetically coupled species with a small coupling constant. Efforts to isolate and characterize this species were unsuccessful.

Mössbauer studies showed that a significant amount of Fe(II) species was present during the catalytic reaction and was slowly converted to the Fe(III) species. This is in contrast to the system studied by DeBeer and co-workers,^30^ where 83% of the Fe(III) N–O bound complex was formed after 15 min, and 66% of the Fe(III) iminyl radical species was formed after 60 min. The difference in iron speciation in these systems suggests that with the addition of the PyBOX ligand, the initial reaction with oxidant is slow relative to the N–O bond cleavage, thus precluding our efforts to observe and isolate an iminyl radical species.

Using the same procedure, the catalytic reaction with 2,2,2-trifluoroethyl (2,4-dichlorobenzoyl)oxycarbamate was studied by ^57^Fe Mössbauer spectroscopy. The freeze-trapped 80 K ^57^Fe Mössbauer spectrum of the catalytic reaction 15 min after oxidant/styrene addition (Figure S6) revealed that just 15% of the iron species is converted to the Fe(III) species, while the remaining iron species are present as high-spin Fe(II) species. Parameters for one of the species (red, Figure S6) are in good agreement with parameters for complex **3**, and it can be observed that this species is converted mostly to the Fe(III) species during the reaction. This indicates that this species is more reactive than other high-spin Fe(II) species, and is completely converted by the end of the reaction. Distribution of species at the end of the reaction is similar to the reaction with *tert*-butyl(2,4-dichlorobenzoyl)oxycarbamate, with 50% of Fe(III) and 50% of Fe(II) species (Figure S7). These findings also suggest that reaction of initially formed Fe(II) species with hydroxylamine is slower, which is in accordance with previous observations (vide infra).

## 3 Conclusions

Analysis of the precatalytic mixture revealed that multiple Fe(II) species were present due to the ligand exchange with solvent molecules, and that the solvent mixture utilized had significant influence on in situ speciation. Isolation of these species and subsequent reactivity studies with the acyloxy carbamate demonstrated that these species show different reactivity and lead to different reaction yields. Additionally, the use of the PyBOX ligand led to the formation of more reactive intermediates than in a previous system reported by DeBeer and co-workers, outlining the effect of a discrete ligand scaffold and the impact on the catalytic performance.

It was also shown that under catalytic conditions no formation of an Fe(IV) nitrene could be observed. Instead, the formation of the seven-coordinate Fe(III) complex with three carboxylates from the functionalized hydroxylamine was observed. This supports the formation of highly reactive intermediates, where just species formed after N–O bond cleavage can be observed in situ. Isolation of the 4,4-dimethyl-1,3-dioxolan-2-iminium in the absence of substrate further supports the formation of highly reactive intermediates. Formation of this species represents a side reaction in the catalytic process and can be prevented by changing terminal group on the carbamate of the functionalized hydroxylamine. Additionally, spin-trap EPR experiments suggested that the reaction proceeds through an intermediate with unpaired electron density on the hydroxylamine nitrogen. Based on the observations in this study and the prior work by DeBeer and co-workers, formation of an iminyl radical species is proposed in this system.

Overall, this study provides unique insight into the iron speciation in iron catalyzed amino-oxygenation of olefins with functionalized hydroxylamines which utilize a bisoxazoline ligand. Isolation of the ring-closed 4,4-dimethyl-1,3-dioxolan-2-iminium from the reaction with *tert*-butyl(2,4-dichlorobenzoyl)oxycarbamate, suggests that the highly reactive radical species undergoes an intramolecular H-atom abstraction and the subsequent C–H amination in the absence of a suitable substrate. This highlights the importance of the identity of the hydroxylamine in these reactions where one could avoid a dead-end pathway by substitution with a substituent devoid of C–H atoms at that position. While the key iron intermediate could not be spectroscopically identified due to its high reactivity in the presence of bisoxazoline ligand, the formation of an iron iminyl radical species as proposed in literature may also be present in this system. This limitation also highlights the importance of the addition of discrete ligands in iron catalyzed amino-oxygenation reactions, where the ligand can help to improve reactivity and broaden substrate scope by controlling reactivity of iron iminyl radical species. Determination of the role of the oxidant and the ligand outlined herein provides the insight necessary to design and improve future methods of iron catalyzed amino-oxygenation reactions to aid in the assembly complex organic molecules.

## Supporting Information Available

Experimental procedures and additional spectra as well as crystal structure reports (PDF)
