# Supplementary material for: Mechanistic Studies of Iron-PyBOX-Catalyzed Olefin Amino-Oxygenation with Functionalized Hydroxylamines
Source: Organometallics. 2023 Apr 28;42(14):1810–7. doi: 10.1021/acs.organomet.3c00067 (PMC10369677; doi:10.1021/acs.organomet.3c00067)
Supplement: Supplementary file 1 — om3c00067_si_001.pdf [file om3c00067_si_001.pdf]

Electronic Supplementary Information for:

Mechanistic Studies of Iron-PyBOX-Catalyzed Olefin Amino-Oxygenation with Functionalised Hydroxylamines

Aleksa Radovic,[‡a] Nikki J. Wolford,[‡a] Hongze Li[b], William W. Brennessel,[a] Hao Xu,[b]* and Michael L. Neidig[a]*

[a]Department of Chemistry, University of Rochester, Rochester, New York 14627, USA

[b]Department of Chemistry, Brandeis University, Waltham, Massachusetts 02453, USA

Table of Contents:

1. Experimental Procedures S2
   1.1. General Considerations S2
   1.2. ^{57}Fe Mössbauer Spectroscopy S2
   1.3. Electron Paramagnetic Resonance (EPR) Spectroscopy S2
   1.4. UV-Vis-NIR Spectroscopy S2
   1.5. Catalytic Reaction Studies S2
   1.6. Synthesis of $Fe(L)(OTf)_2(MeCN)$ (**1**) S3
   1.7. Synthesis of $[Fe(L)_2][OTf]_2$ (**2**) S3
2. Supplementary Data S3
   2.1. Mössbauer Data S3
   2.2. EPR Data S8
   2.3. UV-Vis-NIR Data S11
   2.4. NMR Data S14
3. Single Crystal X-ray Diffraction Data S15
   3.1. $[Fe(L)_2][OTf]_2 \cdot 2DCM$ (**2**) S15
   3.2. $[Fe(L)(MeCN)_3][Na(NTf_2)_3]$ (**3**) S18
   3.3. $Fe(L)(DCB)_3$ (**4**) S21
   3.4. [4,4-dimethyl-1,3-dioxolan-2-iminium][OTf][L] · DCM (**5**) S24
4. References S26

1. Experimental Procedures

1.1. General Considerations – All solvents and substrate were purchased from commercial sources. All air and moisture sensitive manipulations were carried out in an MBraun inert-atmosphere (N_2) dry box equipped with a direct liquid nitrogen inlet line. All anhydrous solvents were further dried using activated alumina/4 Å molecular sieves and stored under N_2-atmosphere over 4 Å molecular sieves. $FeOTf_2$, PyBOX ligand and hydroxylamine were synthesized according to the reported procedures.[1,2] $^{57}FeOTf_2$ was synthesized starting from ^{57}Fe metal (95 % enriched) purchased from Isoflex. Low-temperature reactions (-15 °C) were performed in the glovebox using a Huber ministat 230-cc-NR recirculating bath combined with a Syrris hot/cold plate fit with a PT100 thermocouple for direct measurement and control of the reaction temperature. Quantitative ^{1}H NMR analyses were performed with 1,3,5-trimethoxybenzene as an internal standard, on a Bruker Avance 400 MHz NMR spectrometer at ambient temperature. Elemental analysis data were obtained from the CENTC Elemental Analysis Facility at the University of Rochester. Microanalysis samples were weighed with a PerkinElmer Model AD6000 Autobalance and their compositions were determined with a PerkinElmer 2400 Series II Analyzer. Air-sensitive samples were handled in a VAC Atmospheres glovebox.

1.2. ^{57}Fe Mössbauer Spectroscopy – Solution samples for ^{57}Fe Mössbauer spectroscopy were prepared from mixture of $Fe(OTf)_2$ and $^{57}Fe(OTf)_2$ salts. Solid samples are prepared starting with pure $^{57}Fe(OTf)_2$. All samples were prepared in an N_2 atmosphere dry glovebox equipped with a liquid N_2 fill port to enable sample freezing to 77 K. Each sample was loaded into a Mössbauer sample cup manufactured from Delrin and stored, handled, and loaded under liquid N_2. Low-temperature ^{57}Fe Mössbauer measurements were performed using a See Co. MS4 Mössbauer spectrometer integrated with a Janis SVT-400T He/N_2 cryostat for measurements at 80 K. Isomer shifts were determined relative to α-Fe at 298 K. All Mössbauer spectra were fit using the program WMoss (SeeCo). Errors of the fit analyses were the following: $\delta \pm 0.02$ mm/s and $\Delta E_Q \pm 3\%$. For multicomponent fits, the quantitation errors of individual components were ± 3%.

1.3. Electron Paramagnetic Resonance (EPR) Spectroscopy – All samples for EPR spectroscopy were prepared in an N_2 atmosphere glove box equipped with a liquid N_2 fill port to enable sample freezing to 77 K. EPR samples were prepared in 4 mm OD suprasil quartz EPR tubes from Wilmad Labglass. X-band EPR spectra were recorded on a Bruker EMXplus spectrometer equipped with a 4119HS cavity and an Oxford ESR-900 helium flow cryostat for measurements at temperatures between 3.9 and 100 K. The instrumental parameters employed for all samples were as follows: 1 mW power; modulation amplitude 1 or 8 Gauss; frequency ≈ 9.38 or 9.85 GHz; modulation frequency 100 kHz.

1.4. UV-Vis-NIR Spectroscopy – Spectra were collected using a Cary 6000i UV-Vis-NIR spectrometer fitted with a Unisoku cryostat in an air-tight 1 cm quartz cuvettes.

1.5. Catalytic Reaction Studies – Catalytic reactions studied by EPR spectroscopy were performed under same conditions as reported in literature, and freeze trapped in liquid N_2 at different timepoints during reaction. Catalytic reactions studied by ^{57}Mössbauer spectroscopy were performed in pure MeCN instead of DCM/MeCN mixture due to the incompatibility of this technique with chlorinated solvents. Rest of the conditions were same as in original contribution. Catalytic reactions studied by UV-Vis-NIR spectroscopy were performed by fast addition of $Fe(OTf)_2$ and ligand mixture to the mixture of styrene and

hydroxylamine to keep concentration constant during the experiment. Additionally, concentrations are lowered 10 times. Rest of the conditions were same as in original contribution.

1.6. Synthesis of $Fe(L)(OTf)_2(MeCN)$ (1) – To a 20 mL scintillation vial fitted with a teflon stir bar was added $Fe(OTf)_2$ (14.2 mg, 0.04 mmol), PyBOX ligand (11.0 mg, 0.04 mmol), 0.2 mL of anhydrous MeCN and 1 mL of anhydrous DCM. Light red solution is stirred at room temperature for 20 minutes before filtering through a Celite pad. After a day of slow diffusion of Et_2O in red solution at -30 °C, red crystals suitable for XRD analysis were formed (13.1 mg, 49 % yield). Elemental analysis (C, H, N): Calculated for $C_{19}H_{22}F_6FeN_4O_8S_2$: C - 34.14 %, H - 3.32 %, N - 8.38 %; found: C - 34.15 %, H - 3.26 %, N - 8.23 %.

1.7. Synthesis of $[Fe(L)_2][OTf]_2$ (2) – To a 20 mL scintillation vial fitted with a teflon stir bar was added $Fe(OTf)_2$ (14.2 mg, 0.04 mmol), PyBOX ligand (22.0 mg, 0.08 mmol), and 1 mL of anhydrous DCM. Dark red solution is stirred at room temperature for 20 minutes before filtering through a Celite pad. After a day of slow diffusion of Et_2O in red solution at -30 °C, dark red crystals suitable for XRD analysis were formed (27.0 mg, 75 % yield). Elemental analysis (C, H, N): Calculated for $C_{32}H_{38}F_6FeN_6O_{10}S_2$: 42.68 %, H - 4.25 %, N - 9.33 %; found: 42.67 %, H - 4.09 %, N - 9.19 %.

2. Supplementary Data

2.1. Mössbauer Data

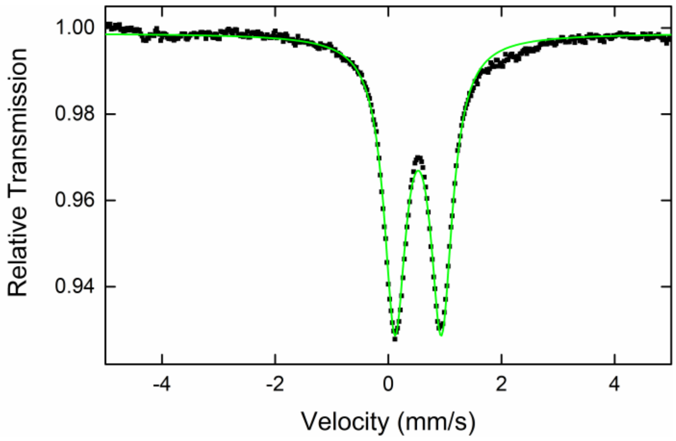

Figure S1. Freeze-trapped 80 K ^{57}Fe Mössbauer spectrum of reaction between complex **1** and tert-butyl (2,4-dichlorobenzoyl)oxycarbamate (8 equivalents).

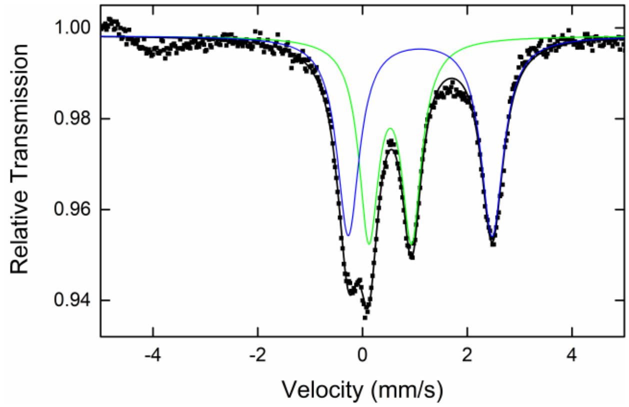

Figure S2. Freeze-trapped 80 K ^{57}Fe Mössbauer spectrum of reaction between complex **2** and tert-butyl (2,4-dichlorobenzoyl)oxycarbamate (8 equivalents) (black line – total fit; blue and green lines – individual components).

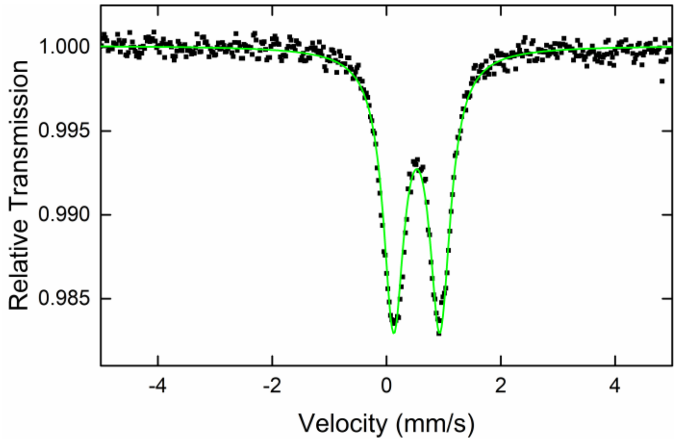

Figure S3. Solid state 80 K ^{57}Fe Mössbauer spectrum of complex **4**.

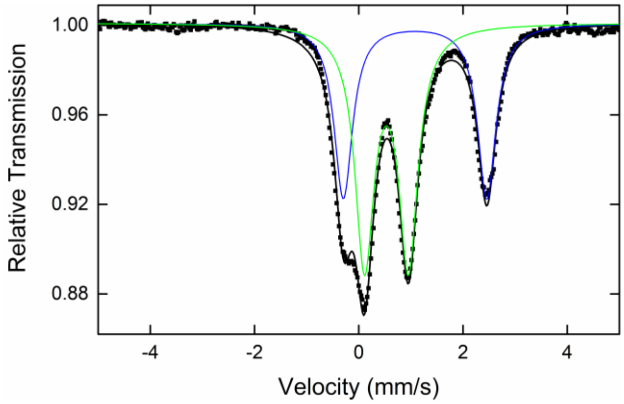

Figure S4. Freeze-trapped 80 K ^{57}Fe Mössbauer spectrum of catalytic reaction with tert-butyl (2,4-dichlorobenzoyl)oxycarbamate, 60 min after beginning of reaction (black line – total fit; blue and green lines – individual components).

Table S1. Percentage of species formed at different timepoints during catalytic reaction with tert-butyl (2,4-dichlorobenzoyl)oxycarbamate, determined by 80 K ^{57}Fe Mössbauer spectroscopy.

Time (min)	**Blue** (%)	**Green** (%)
5	62	38
10	55	45
15	54	46
20	50	50
35	67	53
60	42	58

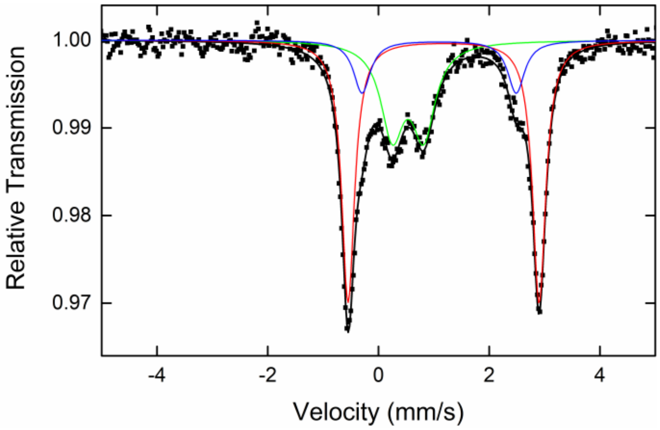

Figure S5. Freeze-trapped 80 K ^{57}Fe Mössbauer spectrum of reaction between pre-catalytic mixture and 2,2,2-Trifluoroethyl (2,4-dichlorobenzoyl)oxycarbamate (12 equivalents) (black line – total fit; blue, red green lines – individual components).

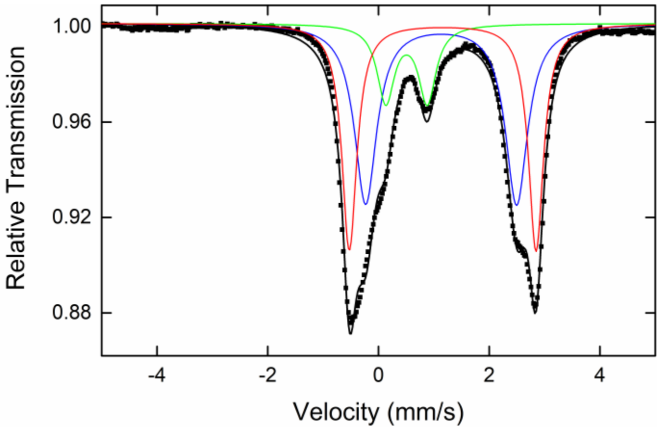

Figure S6. Freeze-trapped 80 K ^{57}Fe Mössbauer spectrum of catalytic reaction with 2,2,2-Trifluoroethyl (2,4-dichlorobenzoyl)oxycarbamate, 15 min after beginning of reaction (black line – total fit; blue, red green lines – individual components).

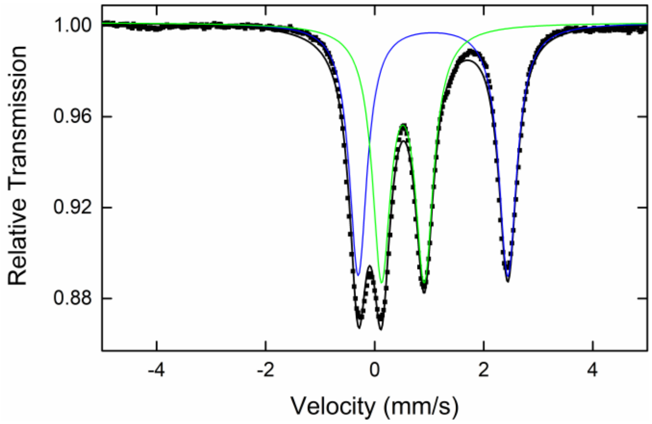

Figure S7. Freeze-trapped 80 K ^{57}Fe Mössbauer spectrum of catalytic reaction with 2,2,2-Trifluoroethyl (2,4-dichlorobenzoyl)oxycarbamate, 60 min after beginning of reaction (black line – total fit; blue and green lines – individual components).

Table S2. Percentage of species formed at different timepoints during catalytic reaction with 2,2,2-Trifluoroethyl (2,4-dichlorobenzoyl)oxycarbamate, determined by 80 K ^{57}Fe Mössbauer spectroscopy.

Time (min)	**Blue** (%)	**Red** (%)	**Green** (%)
5	40	45	15
10	43	43	14
15	46	39	15
20	46	28	26
35	50	6	44
60	50	0	50

2.2. EPR Data

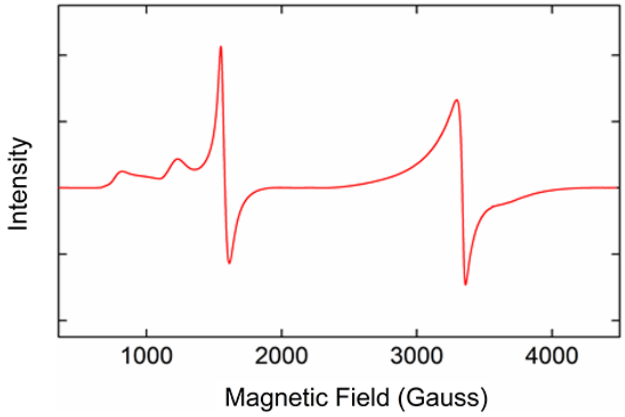

Figure S8. Freeze-trapped 10 K X-band EPR spectra of reaction between pre-catalytic mixture and tert-butyl (2,4-dichlorobenzoyl)oxycarbamate (8 equivalents).

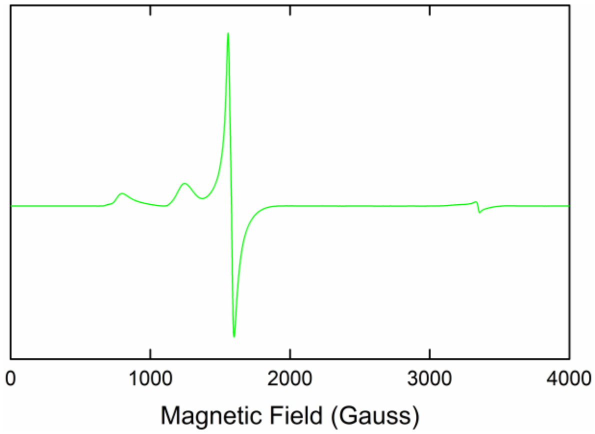

Figure S9. 10 K X-band EPR spectrum of frozen solution of complex **4** in DCM.

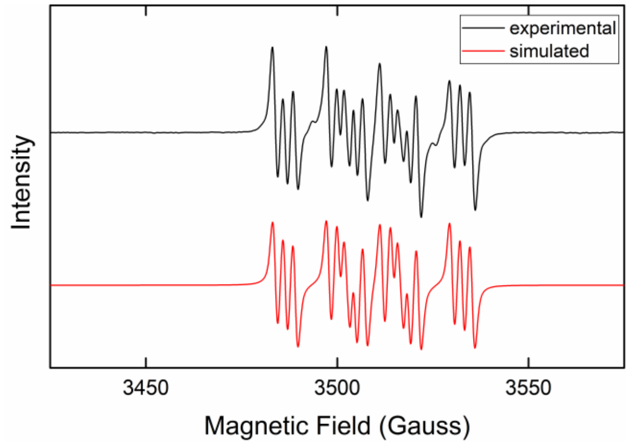

Figure S10. Room temperature X-band EPR spectrum of reaction with DMPO spin trap and 2,2,2-Trifluoroethyl (2,4-dichlorobenzoyl)oxycarbamate (black line) and simulated spectrum (red line). Simulation parameters are g = 2.00670, A_N = 18.2327 G, A_H = 14.0151 G and A_N = 2.59625 G.

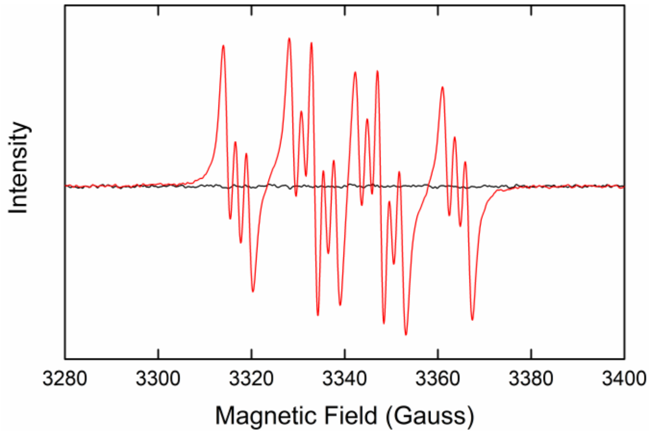

Figure S11. Room temperature X-band EPR spectrum of pre-catalytic mixture with DMPO spin trap (black line) and reaction of pre-catalytic mixture with 2,2,2-Trifluoroethyl (2,4-dichlorobenzoyl)oxycarbamate and DMPO spin trap (red line).

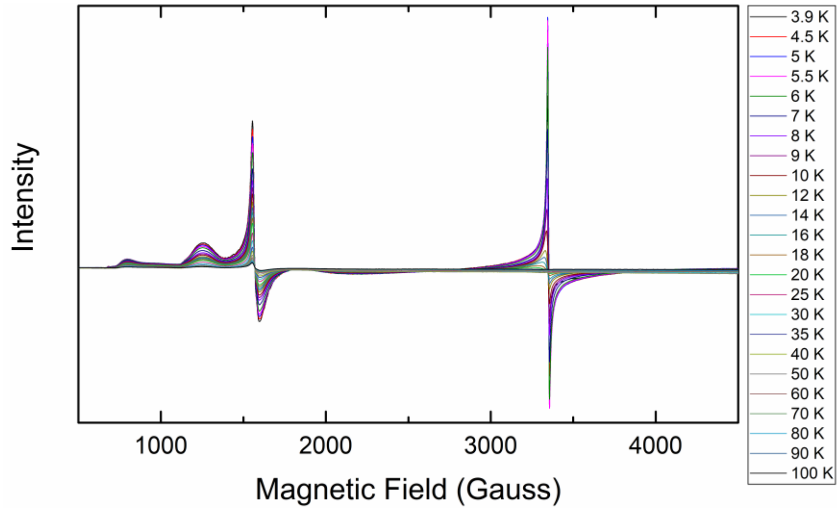

Figure S12. Freeze-trapped X-band EPR spectra of catalytic reaction with tert-butyl (2,4-dichlorobenzoyl)oxycarbamate 5 minutes from beginning reaction at different temperatures.

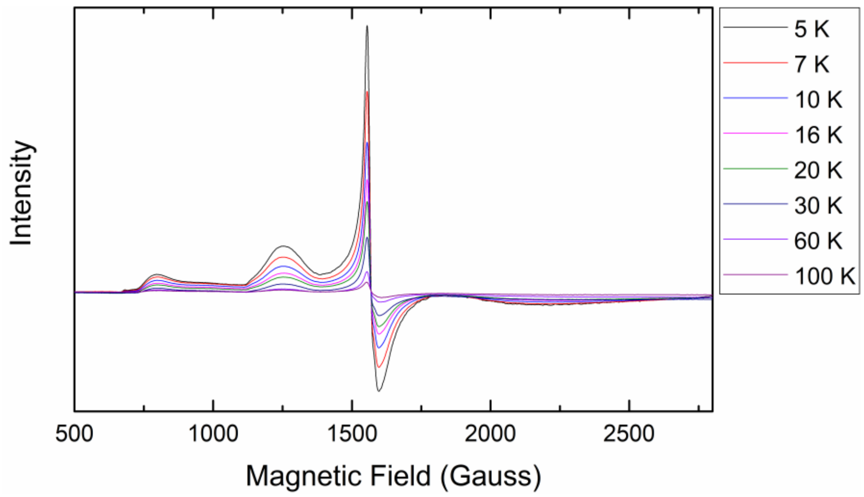

Figure S13. Freeze-trapped X-band EPR spectra of catalytic reaction with tert-butyl (2,4-dichlorobenzoyl)oxycarbamate 5 minutes from beginning reaction showing region of signal at g = 4.25 at different temperatures.

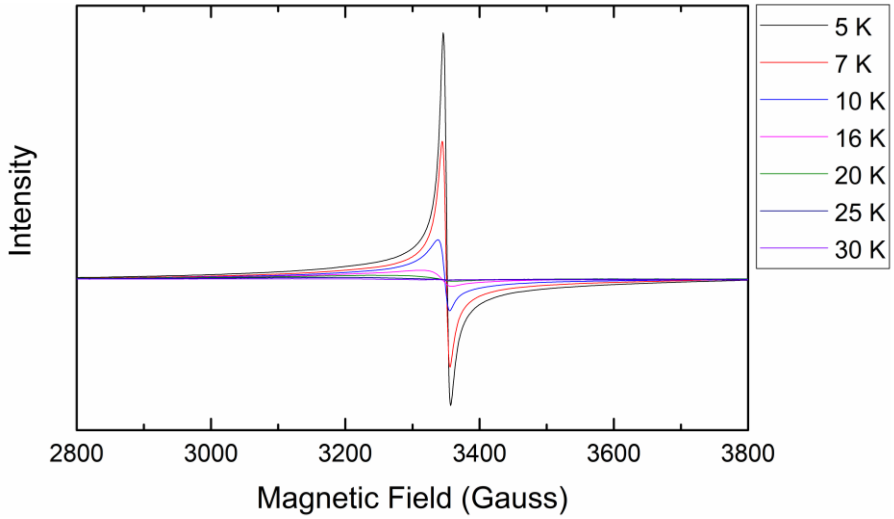

Figure S14. Freeze-trapped X-band EPR spectra of catalytic reaction with tert-butyl (2,4-dichlorobenzoyl)oxycarbamate 5 minutes from beginning reaction showing region of signal at g = 2.01 at different temperatures.

2.3. UV-Vis-NIR Data

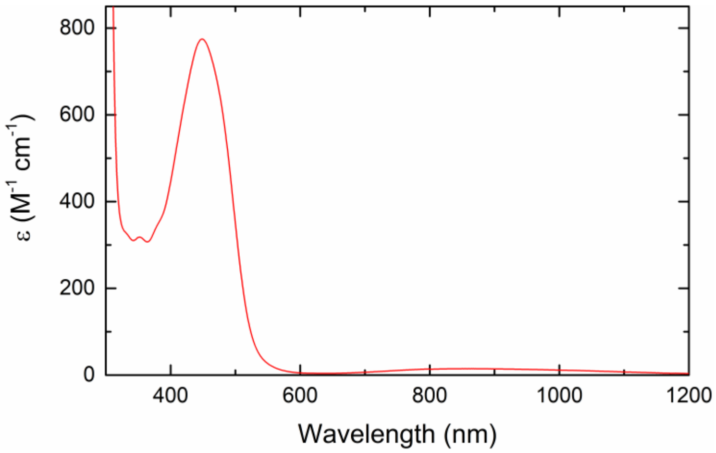

Figure S15. UV-Vis-NIR spectrum of complex **1** in MeCN.

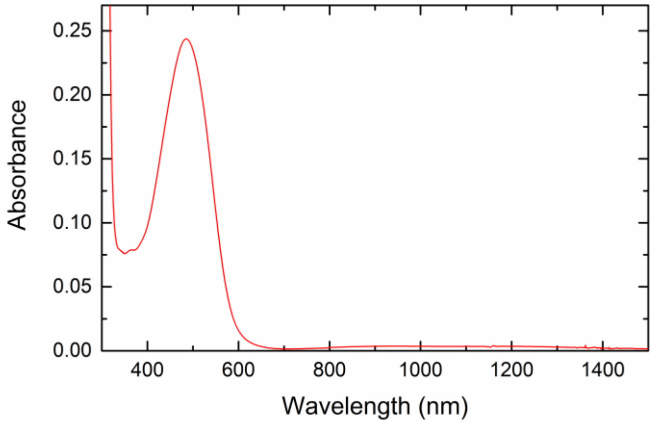

Figure S16. UV-Vis-NIR spectrum of complex **1** in DCM.

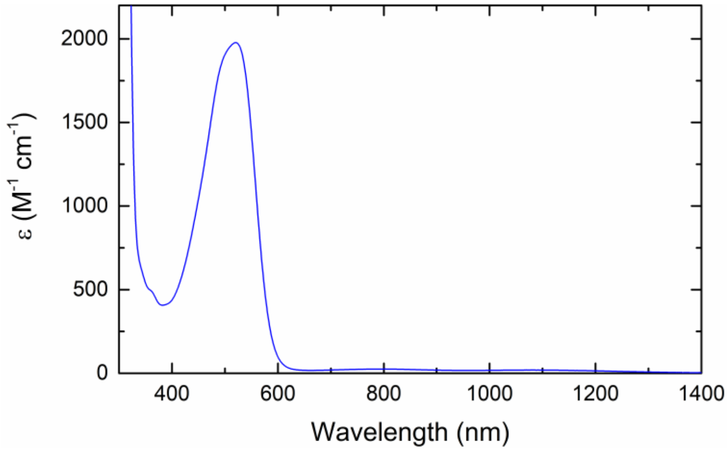

Figure S17. UV-Vis-NIR spectrum of complex **2** in MeCN.

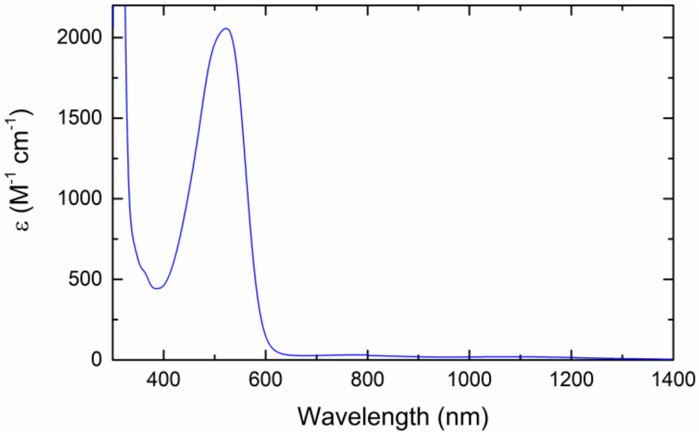

Figure S18. UV-Vis-NIR spectrum of complex **2** in DCM.

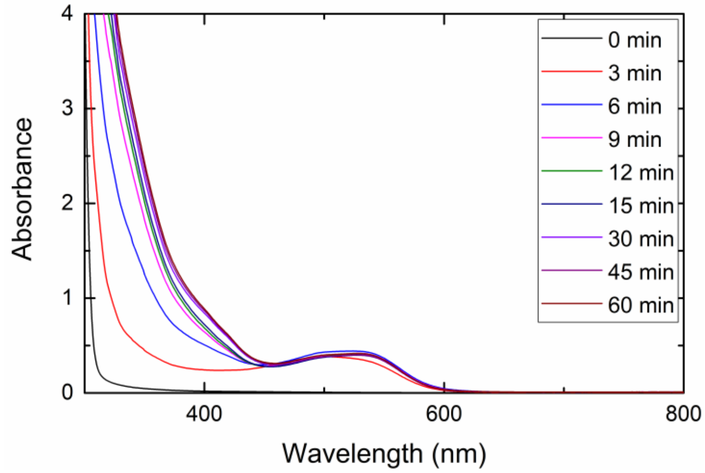

Figure S19. UV-Vis-NIR spectrum of catalytic reaction with tert-butyl (2,4-dichlorobenzoyl)oxycarbamate at different timepoints. Spectrum at timepoint 0 represents spectrum of mixture of hydroxylamine and styrene.

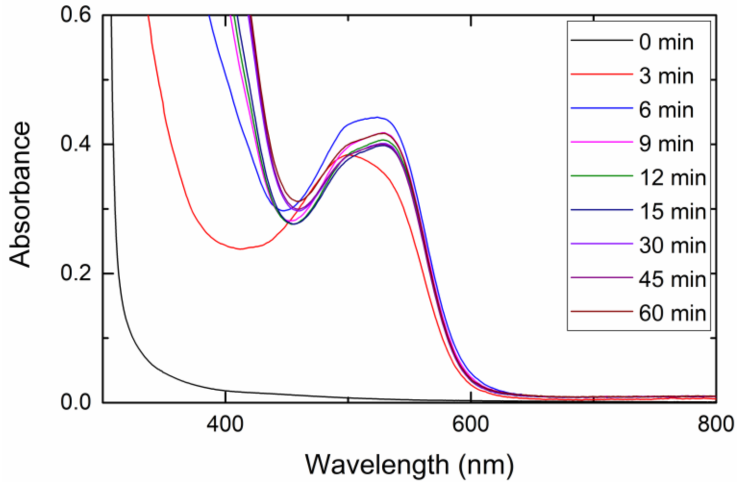

Figure S20. UV-Vis-NIR spectrum of catalytic reaction with tert-butyl (2,4-dichlorobenzoyl)oxycarbamate at different timepoints focusing on low energy transitions.

2.4. NMR Data

Table S3. NMR determined yields of catalytic reactions with tert-butyl (2,4-dichlorobenzoyl)oxycarbamate under different conditions.

Conditions	Yield (%)
DCM/MeCN (20:1), slow addition, -15 °C	48
MeCN, slow addition, -15 °C	29
MeCN, RT	45
Complex **1**, MeCN, RT*	58
Complex **2**, MeCN, RT*	19

*Solutions of complexes 1 and 2 were used instead of pre-catalytic mixture

3. Single Crystal X-ray Diffraction Data

3.1. $[Fe(L)_2][OTf]_2 \cdot 2DCM$ (**2**)

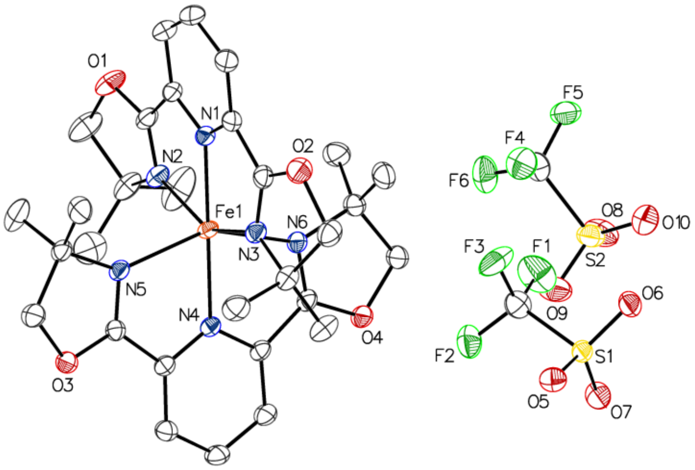

Data collection: A crystal (0.339 x 0.121 x 0.04 mm^3) was placed onto a thin glass optical fiber or a nylon loop and mounted on a Rigaku XtaLAB Synergy-S Dualflex diffractometer equipped with a HyPix-6000HE HPC area detector for data collection at 100.00(10) K. A preliminary set of cell constants and an orientation matrix were calculated from a small sampling of reflections.[3] A short pre-experiment was run, from which an optimal data collection strategy was determined. The full data collection was carried out using a PhotonJet (Cu) X-ray source with frame times of 0.76 and 3.03 seconds and a detector distance of 31.2 mm. Series of frames were collected in 0.50° steps in *w* at different 2*q*, *k*, and *f* settings. After the intensity data were corrected for absorption, the final cell constants were calculated from the xyz centroids of 41352 strong reflections from the actual data collection after integration.[3] See Table S4 for additional crystal and refinement information.

Structure solution and refinement: The structure was solved using SHELXT[4] and refined using SHELXL.[5] The space group *P*-1 was determined based on intensity statistics. Most or all non-hydrogen atoms were assigned from the solution. Full-matrix least squares / difference Fourier cycles were performed which located any remaining non-hydrogen atoms. All non-hydrogen atoms were refined with anisotropic displacement parameters. All hydrogen atoms were placed in ideal positions and refined as riding atoms with relative isotropic displacement parameters. The final full matrix least squares refinement converged to $R1 = 0.0452$ (F^2, $I > 2s(I)$) and $wR2 = 0.1337$ (F^2, all data).

Structure description: The asymmetric unit contains one dicationic iron complex, two triflate anions, and two co-crystallized dichloromethane solvent molecules, all in general positions. One triflate anion and one co-crystallized dichloromethane solvent molecule are modeled as disordered over two positions each (0.56:0.44 and 0.81:0.19, respectively). Structure manipulation and figure generation were performed using Olex2.[6] Unless noted otherwise all structural diagrams containing anisotropic displacement ellipsoids are drawn at the 50 % probability level. Data collection, structure solution, and structure refinement were conducted at the X-ray Crystallographic Facility, B04 Hutchison Hall, Department of Chemistry, University of Rochester.

Table S4. Crystal data and structure refinement for $[Fe(L)_2][OTf]_2 \cdot 2DCM$.

Identification code	2	
Empirical formula	C34 H42 Cl4 F6 Fe N6 O10 S2	
Formula weight	1070.50	
Temperature	100.00(10) K	
Wavelength	1.54184 Å	
Crystal system	triclinic	
Space group	*P*-1	
Unit cell dimensions	*a* = 9.76320(10) Å	a = 91.6660(10)°
	b = 13.68000(10) Å	b = 100.4630(10)°
	c = 17.60040(10) Å	g = 102.4820(10)°
Volume	2251.27(3) $Å^3$	
Z	2	
Density (calculated)	1.579 Mg/m^3	
Absorption coefficient	6.490 mm^{-1}	
F(000)	1096	
Crystal color, morphology	orange-red, plate	
Crystal size	0.339 x 0.121 x 0.04 mm^3	
Theta range for data collection	2.559 to 77.812°	
Index ranges	$-9 \le h \le 12$, $-17 \le k \le 17$, $-22 \le l \le 22$	
Reflections collected	55964	
Independent reflections	9494 [*R*(int) = 0.0599]	
Observed reflections	9090	
Completeness to theta = 74.504°	99.7%	
Absorption correction	Multi-scan	
Max. and min. transmission	1.00000 and 0.37596	
Refinement method	Full-matrix least-squares on F^2	
Data / restraints / parameters	9494 / 240 / 665	
Goodness-of-fit on F^2	1.093	
Final *R* indices [*I*>2sigma(*I*)]	*R*1 = 0.0452, *wR*2 = 0.1326	
R indices (all data)	*R*1 = 0.0463, *wR*2 = 0.1337	
Largest diff. peak and hole	0.613 and -0.815 e.$Å^3$	

Table S5. Selected bond lengths [Å] and angles [°] for $[Fe(L)_2][OTf]_2 \cdot 2DCM$.

Fe(1)-N(1)	2.1033(17)	N(2)-Fe(1)-N(5)	93.69(6)
Fe(1)-N(2)	2.1883(16)	N(2)-Fe(1)-N(6)	96.43(6)
Fe(1)-N(3)	2.2217(16)	N(3)-Fe(1)-N(5)	91.56(6)
Fe(1)-N(4)	2.1004(16)	N(4)-Fe(1)-N(1)	173.34(6)
Fe(1)-N(5)	2.2344(16)	N(4)-Fe(1)-N(2)	110.34(6)
Fe(1)-N(6)	2.2099(16)	N(4)-Fe(1)-N(3)	99.90(6)
N(1)-Fe(1)-N(2)	75.30(7)	N(4)-Fe(1)-N(5)	74.75(6)
N(1)-Fe(1)-N(3)	74.34(6)	N(4)-Fe(1)-N(6)	75.05(6)
N(1)-Fe(1)-N(5)	101.75(6)	N(6)-Fe(1)-N(3)	93.87(6)
N(1)-Fe(1)-N(6)	108.33(6)	N(6)-Fe(1)-N(5)	149.80(6)
N(2)-Fe(1)-N(3)	149.63(6)		

3.2. $[Fe(L)(MeCN)_3][Na(NTf_2)_3]$ (**3**)

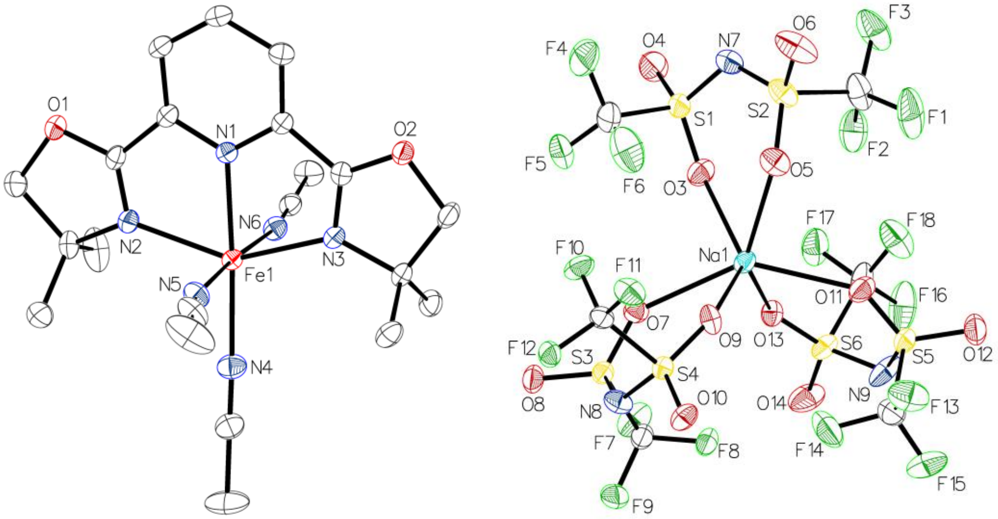

Data collection: A crystal (0.369 x 0.196 x 0.117 mm^3) was placed onto a thin glass optical fiber or a nylon loop and mounted on a XtaLab Synergy-S Dualflex diffractometer equipped with a HyPix-6000HE HPC area detector for data collection at 100.0(1) K. A preliminary set of cell constants and an orientation matrix were calculated from a small sampling of reflections.[3] A short pre-experiment was run, from which an optimal data collection strategy was determined. The full data collection was carried out using a PhotonJet (Cu) X-ray Source with frame times of 0.09 and 0.37 seconds and a detector distance of 31.2 mm. Series of frames were collected in 0.50° *w* at different 2*q*, *k*, and *f* settings. After the intensity data were corrected for absorption, the final cell constants were calculated from the xyz centroids of 24515 strong reflections from the actual data collection after integration.[3] See Table S6 for additional crystal and refinement information.

Structure solution and refinement: The structure was solved using SHELXT[4] and refined using SHELXL.[5] The space group $P2_1/c$ was determined based on systematic absences. Most or all non-hydrogen atoms were assigned from the solution. Full-matrix least squares / difference Fourier cycles were performed which located any remaining non-hydrogen atoms. All non-hydrogen atoms were refined with anisotropic displacement parameters. All hydrogen atoms were placed in ideal positions and refined as riding atoms with relative isotropic displacement parameters. The final full matrix least squares refinement converged to $R1 = 0.0410$ (F^2, $I > 2\sigma(I)$) and $wR2 = 0.1032$ (F^2, all data).

Structure description: The asymmetric unit contains one dicationic iron complex and one $Na(NTf_2)_3$ dianion, both in general positions. Structure manipulation and figure generation were performed using Olex2.[6] Unless noted otherwise all structural diagrams containing thermal displacement ellipsoids are drawn at the 50 % probability level. Data collection, structure solution, and structure refinement were conducted at the X-ray Crystallographic Facility, B04 Hutchison Hall, Department of Chemistry, University of Rochester.

Table S6. Crystal data and structure refinement for $[Fe(L)(MeCN)_3][Na(NTf_2)_3]$.

Identification code	3	
Empirical formula	C27 H28 F18 Fe N9 Na O14 S6	
Formula weight	1315.78	
Temperature	100.0(1) K	
Wavelength	1.54184 Å	
Crystal system	monoclinic	
Space group	$P2_1/c$	
Unit cell dimensions	*a* = 15.26410(10) Å	⍰ = 90°
	b = 11.17160(10) Å	⍰ = 100.0260(10)°
	c = 29.6426(3) Å	⍰ = 90°
Volume	4977.60(8) $Å^3$	
Z	4	
Density (calculated)	1.756 Mg/m^3	
Absorption coefficient	6.122 mm^{-1}	
F(000)	2640	
Crystal color, morphology	yellow-orange, block	
Crystal size	0.369 x 0.196 x 0.117 mm^3	
Theta range for data collection	3.837 to 77.546°	
Index ranges	$-19 \le h \le 17$, $-13 \le k \le 12$, $-35 \le l \le 37$	
Reflections collected	43976	
Independent reflections	10398 [*R*(int) = 0.0374]	
Observed reflections	9732	
Completeness to theta = 74.504°	99.6%	
Absorption correction	Multi-scan	
Max. and min. transmission	1.00000 and 0.14226	
Refinement method	Full-matrix least-squares on F^2	
Data / restraints / parameters	10398 / 0 / 692	
Goodness-of-fit on F^2	1.035	
Final *R* indices [*I*>2sigma(*I*)]	*R*1 = 0.0410, *wR*2 = 0.1000	
R indices (all data)	*R*1 = 0.0454, *wR*2 = 0.1032	
Largest diff. peak and hole	0.680 and -0.582 e.$Å^{-3}$	

Table S7. Selected bond lengths [Å] and angles [°] for $[Fe(L)(MeCN)_3][Na(NTf_2)_3]$.

Fe(1)-N(1)	2.1307(19)	N(3)-Fe(1)-N(5)	87.34(7)
Fe(1)-N(2)	2.1901(19)	N(4)-Fe(1)-N(1)	176.09(8)
Fe(1)-N(3)	2.1843(19)	N(4)-Fe(1)-N(2)	104.00(8)
Fe(1)-N(4)	2.101(2)	N(4)-Fe(1)-N(3)	107.42(8)
Fe(1)-N(5)	2.189(2)	N(4)-Fe(1)-N(5)	86.40(8)
Fe(1)-N(6)	2.159(2)	N(4)-Fe(1)-N(6)	92.00(8)
N(1)-Fe(1)-N(2)	74.32(7)	N(5)-Fe(1)-N(2)	94.68(8)
N(1)-Fe(1)-N(3)	74.33(7)	N(6)-Fe(1)-N(2)	89.68(7)
N(1)-Fe(1)-N(5)	90.22(7)	N(6)-Fe(1)-N(3)	89.23(7)
N(1)-Fe(1)-N(6)	91.51(7)	N(6)-Fe(1)-N(5)	175.60(8)
N(3)-Fe(1)-N(2)	148.59(7)		

3.3. $Fe(L)(DCB)_3$ (**4**)

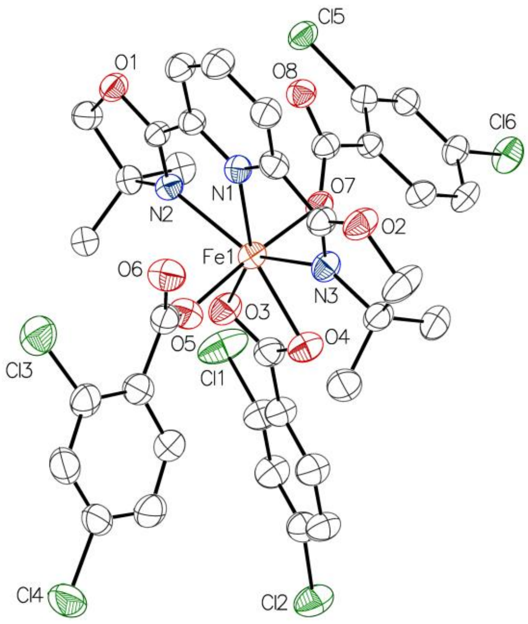

Data collection: A crystal (0.219 x 0.086 x 0.037 mm^3) was placed onto a thin glass optical fiber or a nylon loop and mounted on a Rigaku XtaLAB Synergy-S Dualflex diffractometer equipped with a HyPix-6000HE HPC area detector for data collection at 100.00(10) K. A preliminary set of cell constants and an orientation matrix were calculated from a small sampling of reflections.[3] A short pre-experiment was run, from which an optimal data collection strategy was determined. The full data collection was carried out using a PhotonJet (Cu) X-ray source with frame times of 7.58 and 36.32 seconds and a detector distance of 31.2 mm. Series of frames were collected in 0.50º steps in *w* at different 2*q*, *k*, and *f* settings. After the intensity data were corrected for absorption, the final cell constants were calculated from the xyz centroids of 11885 strong reflections from the actual data collection after integration.[3] See Table S8 for additional crystal and refinement information.

Structure solution and refinement: The structure was solved using SHELXT[4] and refined using SHELXL.[5] The space group $P2_1/c$ was determined based on systematic absences. Most or all non-hydrogen atoms were assigned from the solution. Full-matrix least squares / difference Fourier cycles were performed which located any remaining non-hydrogen atoms. All non-hydrogen atoms were refined with anisotropic displacement parameters. All hydrogen atoms were placed in ideal positions and refined as riding atoms with relative isotropic displacement parameters. The final full matrix least squares refinement converged to $R1 = 0.0743$ (F^2, $I > 2s(I)$) and $wR2 = 0.2347$ (F^2, all data).

Structure description: The asymmetric unit contains one molecule in a general position. Structure manipulation and figure generation were performed using Olex2.[6] Unless noted otherwise all structural diagrams containing anisotropic displacement ellipsoids are drawn at the 50 % probability level. Data collection, structure solution, and structure refinement were conducted at the X-ray Crystallographic Facility, B04 Hutchison Hall, Department of Chemistry, University of Rochester.

Table S8. Crystal data and structure refinement for $Fe(L)(DCB)_3$.

Identification code	4	
Empirical formula	C36 H28 Cl6 Fe N3 O8	
Formula weight	899.16	
Temperature	100.00(10) K	
Wavelength	1.54184 Å	
Crystal system	monoclinic	
Space group	$P2_1/c$	
Unit cell dimensions	*a* = 22.1638(8) Å	a = 90°
	b = 11.0379(4) Å	b = 98.580(3)°
	c = 15.5004(5) Å	g = 90°
Volume	3749.6(2) $Å^3$	
Z	4	
Density (calculated)	1.593 Mg/m^3	
Absorption coefficient	7.643 mm^{-1}	
F(000)	1828	
Crystal color, morphology	colourless, plate	
Crystal size	0.219 x 0.086 x 0.037 mm^3	
Theta range for data collection	4.034 to 77.831°	
Index ranges	$-26 \le h \le 28$, $-13 \le k \le 13$, $-19 \le l \le 18$	
Reflections collected	38772	
Independent reflections	7830 [*R*(int) = 0.0937]	
Observed reflections	6380	
Completeness to theta = 74.504°	99.7%	
Absorption correction	Multi-scan	
Max. and min. transmission	1.00000 and 0.58893	
Refinement method	Full-matrix least-squares on F^2	
Data / restraints / parameters	7830 / 0 / 491	
Goodness-of-fit on F^2	1.101	
Final *R* indices [*I*>2sigma(*I*)]	*R*1 = 0.0743, *wR*2 = 0.2219	
R indices (all data)	*R*1 = 0.0858, *wR*2 = 0.2347	
Largest diff. peak and hole	0.927 and -0.922 e.$Å^{-3}$	

Table S9. Selected bond lengths [Å] and angles [°] for $Fe(L)(DCB)_3$.

Fe(1)-O(3)	2.156(3)	O(5)-Fe(1)-O(3)	86.34(14)
Fe(1)-O(4)	2.163(3)	O(5)-Fe(1)-O(4)	87.11(14)
Fe(1)-O(5)	1.968(3)	O(5)-Fe(1)-N(1)	94.45(14)
Fe(1)-O(7)	1.955(3)	O(5)-Fe(1)-N(2)	92.10(14)
Fe(1)-N(1)	2.209(4)	O(5)-Fe(1)-N(3)	90.88(14)
Fe(1)-N(2)	2.281(4)	O(7)-Fe(1)-O(3)	88.10(13)
Fe(1)-N(3)	2.284(4)	O(7)-Fe(1)-O(4)	87.62(13)
O(3)-Fe(1)-O(4)	60.16(13)	O(7)-Fe(1)-O(5)	173.73(14)
O(3)-Fe(1)-N(1)	150.34(13)	O(7)-Fe(1)-N(1)	91.81(13)
O(3)-Fe(1)-N(2)	78.85(13)	O(7)-Fe(1)-N(2)	89.67(12)
O(3)-Fe(1)-N(3)	138.49(13)	O(7)-Fe(1)-N(3)	91.36(13)
O(4)-Fe(1)-N(1)	149.49(14)	N(1)-Fe(1)-N(2)	71.48(13)
O(4)-Fe(1)-N(2)	138.98(13)	N(1)-Fe(1)-N(3)	71.17(14)
O(4)-Fe(1)-N(3)	78.35(14)	N(2)-Fe(1)-N(3)	142.65(14)

3.4. [4,4-dimethyl-1,3-dioxolan-2-iminium][OTf][L] · DCM (**5**)

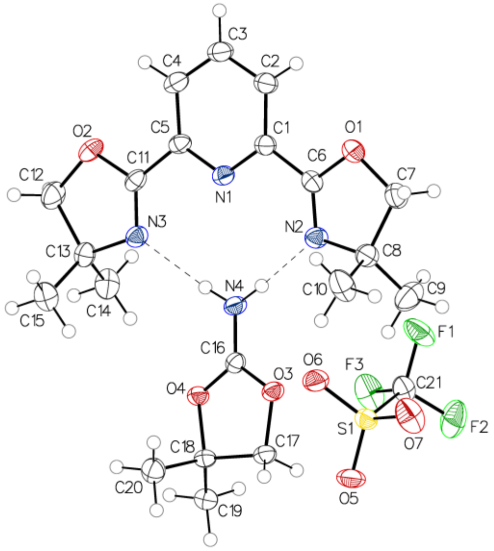

Data collection: A crystal (0.174 x 0.158 x 0.066 mm^3) was placed onto a thin glass optical fiber or a nylon loop and mounted on a Rigaku XtaLAB Synergy-S Dualflex diffractometer equipped with a HyPix-6000HE HPC area detector for data collection at 100.00(10) K. A preliminary set of cell constants and an orientation matrix were calculated from a small sampling of reflections.[3] A short pre-experiment was run, from which an optimal data collection strategy was determined. The full data collection was carried out using a PhotonJet (Cu) X-ray source with frame times of 0.17 and 0.69 seconds and a detector distance of 31.2 mm. Series of frames were collected in 0.50º steps in *w* at different 2*q*, *k*, and *f* settings. After the intensity data were corrected for absorption, the final cell constants were calculated from the xyz centroids of 20883 strong reflections from the actual data collection after integration.[3] See Table S10 for additional crystal and refinement information.

Structure solution and refinement: The structure was solved using SHELXT[4] and refined using SHELXL.[5] The space group $P2_1/c$ was determined based on systematic absences. Most or all non-hydrogen atoms were assigned from the solution. Full-matrix least squares / difference Fourier cycles were performed which located any remaining non-hydrogen atoms. All non-hydrogen atoms were refined with anisotropic displacement parameters. The NH_2 hydrogen atoms were found from the difference Fourier map and refined freely. All other hydrogen atoms were placed in ideal positions and refined as riding atoms with relative isotropic displacement parameters. The final full matrix least squares refinement converged to $R1 = 0.0461$ (F^2, $I > 2s(I)$) and $wR2 = 0.1274$ (F^2, all data).

Structure description: The asymmetric unit contains all species in general positions. Hydrogen bonding links two of the molecules. Structure manipulation and figure generation were performed using Olex2.[6] Unless noted otherwise all structural diagrams containing anisotropic displacement ellipsoids are drawn at the 50 % probability level. Data collection, structure solution, and structure refinement were conducted at the X-ray Crystallographic Facility, B04 Hutchison Hall, Department of Chemistry, University of Rochester.

Table S10. Crystal data and structure refinement for [4,4-dimethyl-1,3-dioxolan-2-iminium][OTf][L] · DCM.

Identification code	5	
Empirical formula	C22 H31 Cl2 F3 N4 O7 S	
Formula weight	623.47	
Temperature	100.00(10) K	
Wavelength	1.54184 Å	
Crystal system	monoclinic	
Space group	$P2_1/c$	
Unit cell dimensions	a = 14.6179(2) Å	a = 90°
	b = 17.0665(3) Å	b = 97.7090(10)°
	c = 11.6463(2) Å	g = 90°
Volume	2879.22(8) $Å^3$	
Z	4	
Density (calculated)	1.438 Mg/m^3	
Absorption coefficient	3.301 mm^{-1}	
F(000)	1296	
Crystal color, morphology	colourless, plate	
Crystal size	0.174 x 0.158 x 0.066 mm^3	
Theta range for data collection	3.051 to 79.094°	
Index ranges	$-18 \le h \le 18$, $-21 \le k \le 21$, $-12 \le l \le 14$	
Reflections collected	48233	
Independent reflections	6078 [R(int) = 0.0831]	
Observed reflections	5113	
Completeness to theta = 74.504°	99.9%	
Absorption correction	Multi-scan	
Max. and min. transmission	1.00000 and 0.50894	
Refinement method	Full-matrix least-squares on F^2	
Data / restraints / parameters	6078 / 39 / 394	
Goodness-of-fit on F^2	1.085	
Final R indices [I>2sigma(I)]	$R1$ = 0.0461, $wR2$ = 0.1204	
R indices (all data)	$R1$ = 0.0548, $wR2$ = 0.1274	
Largest diff. peak and hole	0.326 and -0.498 e.$Å^{-3}$	

Table S11. Selected bond lengths [Å] and angles [°] for [4,4-dimethyl-1,3-dioxolan-2-iminium][OTf][L] · DCM.

O(3)-C(16)	1.307(2)	C(16)-O(4)-C(18)	107.77(14)
O(3)-C(17)	1.457(2)	H(4A)-N(4)-H(4B)	121(2)
O(4)-C(16)	1.309(2)	C(16)-N(4)-H(4A)	117.7(17)
O(4)-C(18)	1.498(2)	C(16)-N(4)-H(4B)	119.4(18)
N(4)-H(4A)	0.91(3)	O(3)-C(16)-O(4)	115.72(16)
N(4)-H(4B)	0.88(3)	N(4)-C(16)-O(3)	121.15(18)
N(4)-C(16)	1.286(2)	N(4)-C(16)-O(4)	123.10(17)
C(17)-C(18)	1.528(3)	O(3)-C(17)-C(18)	104.02(14)
C(16)-O(3)-C(17)	107.75(14)	O(4)-C(18)-C(17)	100.83(14)

4. References

(1) Lu, D. F.; Zhu, C. L.; Jia, Z. X.; Xu, H. Iron(II)-catalyzed intermolecular amino-oxygenation of olefins through the N-O bond cleavage of functionalized hydroxylamines. *J. Am. Chem. Soc.* **2014**, *136* (38), 13186-13189.

(2) Hagen, K. S.; Iron(II) Triflate Salts as Convenient Substitutes for Perchlorate Salts: Crystal Structures of $[Fe(H_2O)_6](CF_3SO_3)_2$ and $Fe(MeCN)_4(CF_3SO_3)_2$. *Inorg. Chem.* **2000**, 39 (25), 5867–5869.

(3) *CrysAlisPro*, version 171.40.84a; Rigaku Corporation: Oxford, UK, **2020**.

(4) Sheldrick, G. M. *SHELXT*, version 2018/2; *Acta. Crystallogr.* **2015**, *A71*, 3-8.

(5) Sheldrick, G. M. *SHELXL*, version 2018/3; *Acta. Crystallogr.* **2015**, *C71*, 3-8.

(6) Dolomanov, O. V.; Bourhis, L. J.; Gildea, R. J.; Howard, J. A. K.; Puschmann, H. *Olex2*, version 1.3-ac4; *J. Appl. Cryst.* **2009**, *42*, 339-341.
